# Temperature during early development has long-term effects on microRNA expression in Atlantic cod

**DOI:** 10.1186/s12864-015-1503-7

**Published:** 2015-04-17

**Authors:** Teshome Tilahun Bizuayehu, Steinar D Johansen, Velmurugu Puvanendran, Hilde Toften, Igor Babiak

**Affiliations:** University of Nordland, Faculty of Biosciences and Aquaculture, Post Box 1490, 8049 Bodø, Norway; Arctic University of Norway, FHS, RNA Lab, Dept Med Biol, N-9037 Tromsø, Norway; Nofima AS, Muninbakken 9-13, P.O. box 6122, NO 9291 Tromsø, Norway

**Keywords:** Atlantic cod, Embryonic development, Methylation, miRNA, Thermal plasticity

## Abstract

**Background:**

Environmental temperature has serious implications in life cycle of aquatic ectotherms. Understanding the molecular mechanisms of temperature acclimation and adaptation of marine organisms is of the uttermost importance for ecology, fisheries, and aquaculture, as it allows modeling the effects of global warming on population dynamics. Regulatory molecules are major modulators of acclimation and adaptation; among them, microRNAs (miRNAs) are versatile and substantial contributors to regulatory networks of development and adaptive plasticity. However, their role in thermal plasticity is poorly known. We have asked whether the temperature and its shift during the early ontogeny (embryonic and larval development) affect the miRNA repertoire of Atlantic cod (*Gadus morhua*), and if thermal experience has long-term consequences in the miRNA profile.

**Results:**

We characterized miRNA during different developmental stages and in juvenile tissues using next generation sequencing. We identified 389 putative miRNA precursor loci, 120 novel precursor miRNAs, and 281 mature miRNAs. Some miRNAs showed stage- or tissue-enriched expression and miRNAs, such as the miR-17 ~ 92 cluster, myomiRs (miR-206), neuromiRs (miR-9, miR-124), miR-130b, and miR-430 showed differential expression in different temperature regimes. Long-term effect of embryonic incubation temperature was revealed on expression of some miRNAs in juvenile pituitary (miR-449), gonad (miR-27c, miR-30c, and miR-200a), and liver (let-7 h, miR-7a, miR-22, miR-34c, miR-132a, miR-192, miR-221, miR-451, miR-2188, and miR-7550), but not in brain. Some of differentially expressed miRNAs in the liver were confirmed using LNA-based rt-qPCR. The effect of temperature on methylation status of selected miRNA promoter regions was mostly inconclusive.

**Conclusions:**

Temperature elevation by several degrees during embryonic and larval developmental stages significantly alters the miRNA profile, both short-term and long-term. Our results suggest that a further rise in seas temperature might affect life history of Atlantic cod.

**Electronic supplementary material:**

The online version of this article (doi:10.1186/s12864-015-1503-7) contains supplementary material, which is available to authorized users.

## Background

According to Intergovernmental Panel on Climate Change (IPCC), elevated level of greenhouse gases is one of the primary causes of global warming effect, manifested in rise in sea level by 0.19 m per decade, including an increase of the upper 75 m by 0.11°C, and changes in local salinity. These changes are predicted to accelerate if no action is taken [1]. Changes in habitat temperature have profound effects on life history of fish. The rising sea temperature is predicted to alter the biodynamics of marine organisms, since the temperature accounts for development, growth, recruitment, maturity, distribution and survival of aquatic ectotherms [2-5]. In economical consequence, it will reduce fisheries and cause a geographic shift of aquaculture [6]. Atlantic cod (*Gadus morhua*) is one of the most important species of northern Atlantic Ocean, both in term of ecology and economy. It has a wide distribution along the coastline of the western Atlantic Ocean up north of North Carolina, eastern Atlantic Ocean up north from the Bay of Biscay, and it inhabits open waters of Arctic Ocean, Barents Sea, Labrador Sea, and coastal waters of Greenland. Populations of Atlantic cod follow seasonal migrations, as well as diurnal migration through different layers of a stratified water column in coastal areas, indicating broad temperature adaptation [7]. On the other hand, Atlantic cod shows high sensitivity to winter warming during the reproductive season [8] and its embryos have rather a narrow thermal window (4–10°C) [9] as compared with juveniles and adults (1–17°C) [2,10]. In spring, water temperature above 8°C can affect Atlantic cod recruitment [11]. Le Bris et al. [12] hypothesized that early-life growth rates might influence migratory behavior of adult cod.

Temperature is a major determinant of physiological processes. Animals evolved behavioral strategies to cope with the recurrent change in ambient temperature, which affects cellular and molecular mechanisms [13]. On the molecular level, regulatory control and response to temperature change can occur at transcriptional, posttranscriptional, translational, and posttranslational levels [14]. To predict the effects of global warming on marine species, molecular mechanisms of acclimation and adaptation to temperature changes need a better understanding. Temperature is among the major factors that determine selection of aquaculture sites, breeding programs, and management strategies. Cultured fish adjust to the farming environment within their physiological limits. Thus, understanding the regulatory mechanism of this adjustment is important for the future success of aquaculture industry in a changing climate. Also, knowledge on mechanisms of physiological response to temperature change can improve fish stock projection models [3].

MicroRNAs (miRNAs) are small regulatory molecules mainly involved in post-transcriptional gene regulation, largely through translational inhibition and degradation of mRNA [15,16]. As regulators of transcriptome, miRNAs likely have a significant role in adaptation of organisms to a changing environment. Temperature during early stage of the life cycle has persistent effects on the expression patterns of a number of genes and consequently, fish growth [17]. Such the thermal imprinting has been reported in different fish species [18-20]. The effect of temperature on miRNA expression has been demonstrated in few fish species [21,22]. However, the relation between sea temperature elevation and miRNA expression has not yet been reported in a marine species. Also, there is lack of knowledge about possible long-term effects of thermal experience during early ontogeny on miRNA expression.

Here, we ask whether the elevated temperature experienced during early life stages affects miRNA expression in Atlantic cod, a cold water marine teleost. Also, we ask whether the temperature during early ontogeny shows long-term effects on miRNA repertoire and has epigenetic footprints in miRNA promoter regions. We reared embryos and larvae under two temperature regimes, 4°C or 9.5°C, either continuously or shifted the temperature during the larval development. The temperature difference reflects the maximum predicted change in sea temperature (1–4°C) by year 2050 [1]. As no systematic data are available on miRNA repertoire in Atlantic cod, we sequenced the small RNA constituents using SOLiD next generation sequencing platform and characterized miRNAs of Atlantic cod during its development. We found that embryonic and/or larval thermal experience has altered miRNA profiles during the development, and that long-term effects are manifested by differential miRNA expression in juvenile tissues.

## Results

### Mapping and characterization of miRNA during the development of Atlantic cod

We obtained over half a billion sequences from small RNA libraries (Table 1). From the total 393,182,014 filtered high quality sequences, 35% were conserved miRNAs, constituting 3.3 million miRNA sequences per library on average. Around 43.2% of the sequences obtained from developmental stages and 53.9% of the sequences obtained from tissues were mapped to the Atlantic cod genome without mismatches. The size distribution of these sequences showed a clear peak at 22 nts (Additional file 1).

**Table 1:** **Summary of sequence counts from small RNA libraries. Samples were obtained from different developmental stages and four tissues (brain, gonad, liver, and pituitary) of Atlantic cod**

**Description**	**Development count**	**Tissues count**
Raw SOLiD reads	303 165 498	268 844 467
Adapter only sequences	676 039	605 210
Reads without 3’ adapter sequence	42 928 670	67 064 659
Low quality sequences	24 440 083	27 931 725
Size ≤ 15 nt	10 286 414	4 750 802
Artifact sequences	83 923	60 426
High quality, filtered sequences	224 750 369	168 431 645
Unique sequences	23 084 734	24 341 799
miRBase v20 alignment		
No mismatches	49 845 256	67 205 304
1 mismatches	9 025 096	11 872 566
Types of conserved mature miRNAs	281	281

Mapping of our sequences to the Atlantic cod genome resulted in many conserved and previously uncharacterized miRNA loci. We identified 389 putative miRNA precursor loci, which included 175 conserved and 120 novel precursor miRNAs. Fifty-two loci had multiple locations in the assembled Atlantic cod genome (Additional file 2). Within the conserved precursors, we identified 281 types of mature miRNAs, which were categorized into 184 miRNA families (Additional file 2).

### Characterization of miRNAs in Atlantic cod

**Figure 1:**
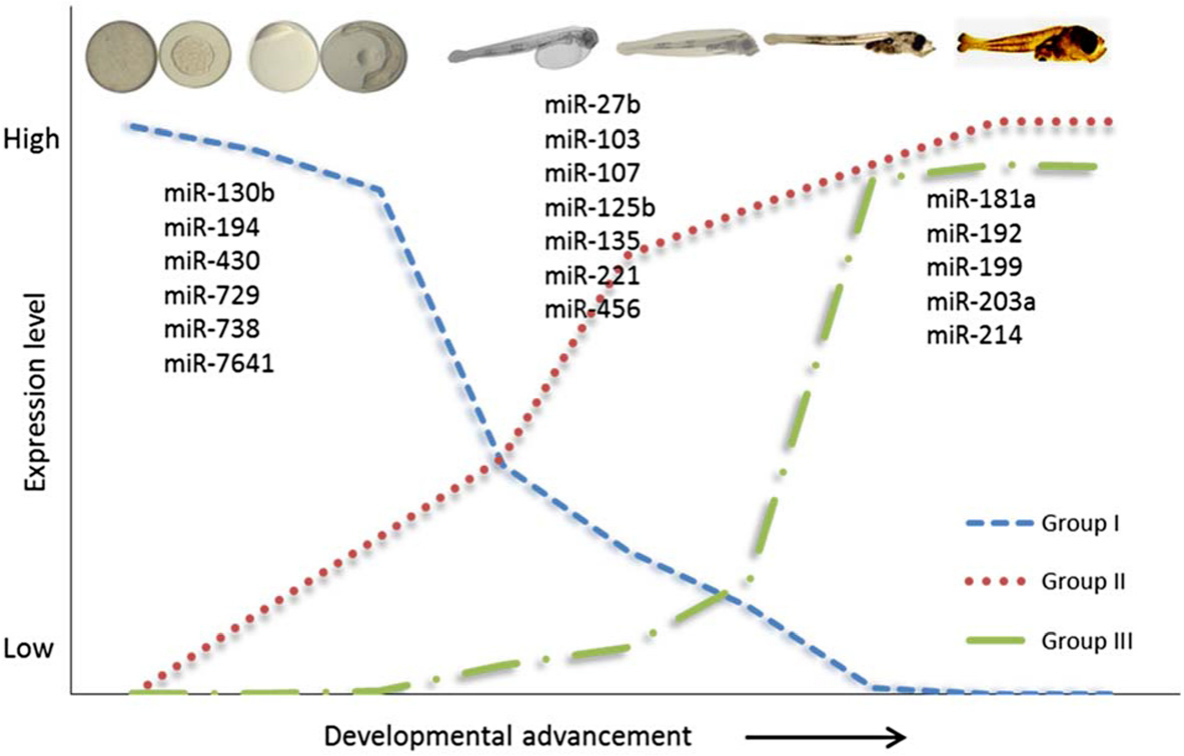
Schematic representation of expression patterns of miRNAs during early development of Atlantic cod. Group I miRNAs are highly expressed before and during MZT and then decrease; Group II miRNAs are highly expressed during organogenesis; and Group III miRNAs are highly expressed during the transition from larval to adult forms. Only selected miRNAs are depicted; see Additional file 3 for the full set of miRNAs.

Characterization of miRNAs during the development was performed using sequences obtained from LL group, which represented a default natural temperature conditions. Many miRNAs showed differential expression among investigated developmental stages. Clustering of miRNAs based on the expression pattern during the development resulted in 3 groups: miRNAs relatively highly expressed before and during maternal-zygotic-transition, MZT (Group I), during organogenesis (Group II), and during metamorphosis (Group III) (Figure 1 and Additional file 3).

**Figure 2:**
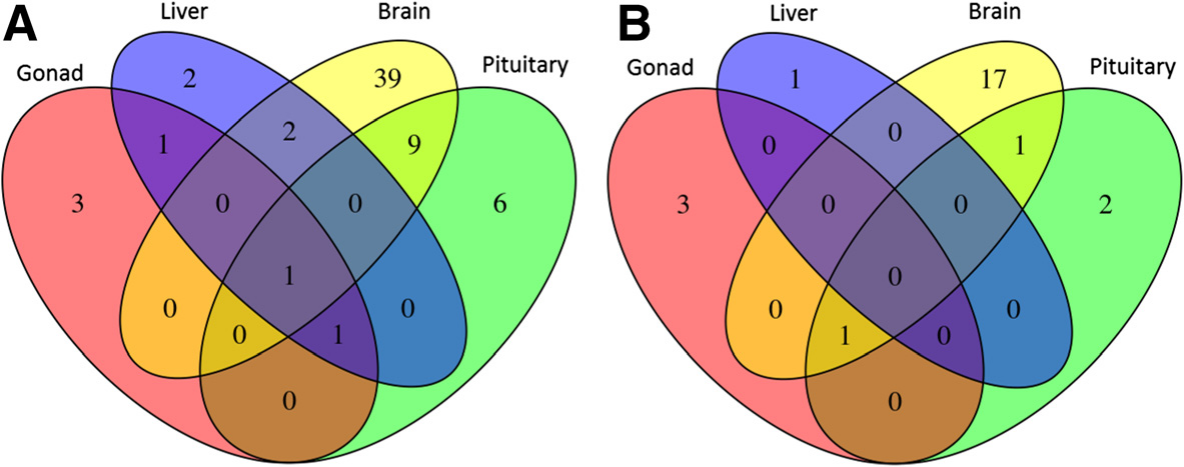
Number of differentially expressed miRNAs between Atlantic cod tissues. Pairwise comparison between different tissues using NOISeq with probability of differential expression **A)** q > 0.95 and **B)** q > 0.99.

Although the diversity of miRNAs among tissues was comparable, their expression patterns were subdivided into 4 dendrograms in a tissue-specific manner regardless of temperature regimes (Additional file 4). Number of differentially expressed miRNAs varied among tissues (Figure 2). Some miRNAs were specifically abundant in a given tissue type; for example, miR-9, miR-124a, and miR-128 in the brain; miR-375 and miR-7a in the pituitary; miR-202 in gonads; and miR-122 in the liver (Additional files 4 and 5).

### Effect of water temperature on miRNA expression in embryos and larvae

**Figure 3:**
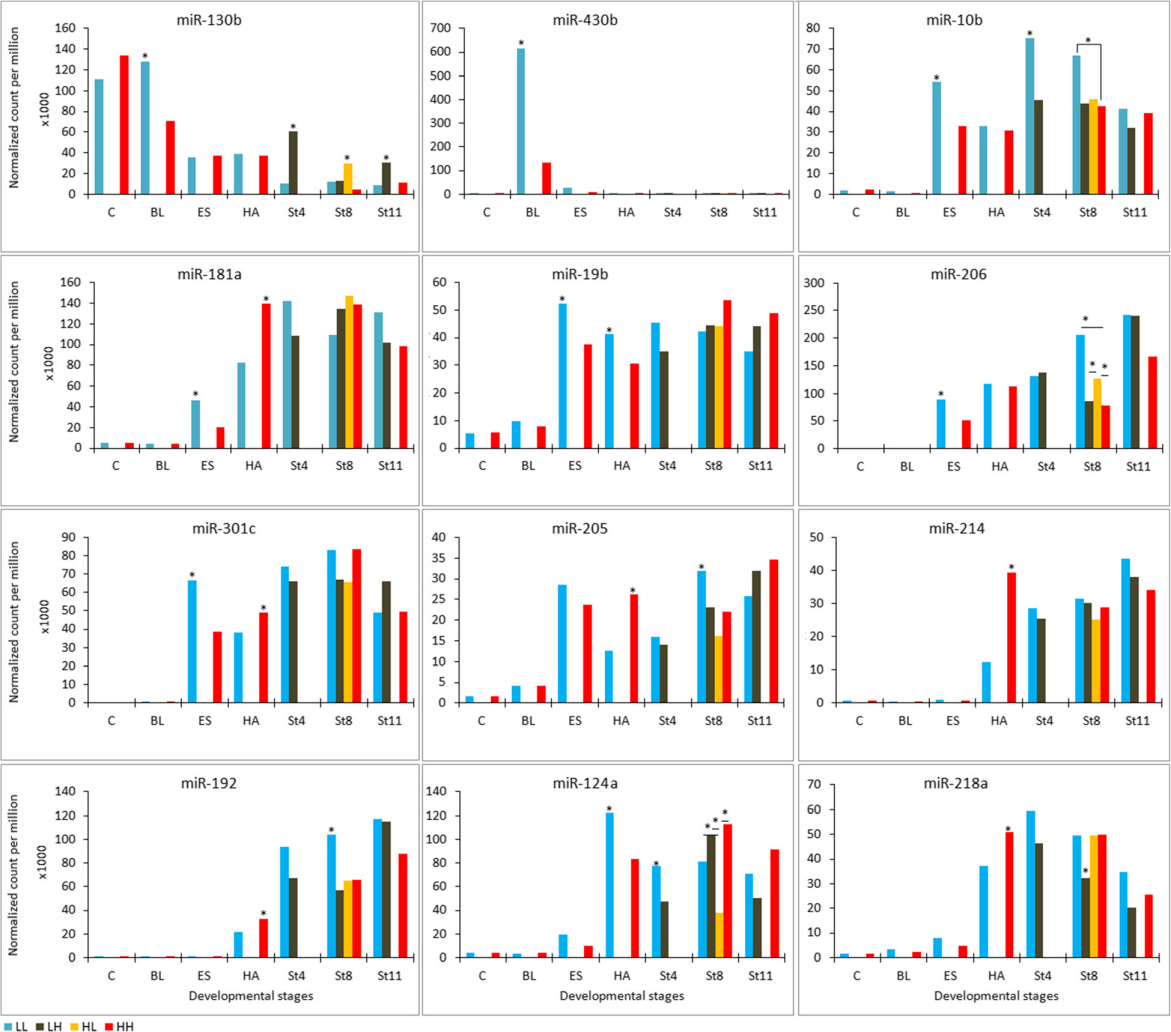
Differential expression of miRNAs during Atlantic cod embryonic development under four temperature regimes, based on pair-wise comparison between treatments. *indicates significant q-value, which is the probability of differential expression, > 0.99. LL, LH, HL, and HH are temperature regimes described in Methods. C, BL, ES, HA, St4, St8, and St11 represent cleavage, blastula, early somitogenesis, hatch, stage 4 larvae, stage 8 larvae, and stage 11 larvae developmental stages, respectively.

The expression levels of 15 miRNA families (miRNAs that share ‘seed’ sequence) were altered by the change in water temperature (Figure 3 and Additional file 6). During the late blastula stage, the transcript level of miR-130b and miR-430 was lower in embryos incubated at 9.5°C than at 4°C (Figure 3). Similarly, other miRNAs (miR-17, miR-18c, miR-19b, miR-20a, miR-103a, miR-130c, miR-181a, miR-206, and miR-301c) had lower abundance in embryos incubated at 9.5°C during early somite formation. At hatch, the expression level of 12 miRNAs (miR-99, miR-103, miR-125b, miR-181a, miR-181b, miR-192, miR-203a, miR-204, miR-205, miR-214, miR-218a, and miR-301c) was higher, and 4 miRNAs (miR-9, miR-19a, miR-19b, and miR-124) was lower in embryos incubated at 9.5°C compared to 4°C (Figure 3 and Additional file 6).

During larval development, 14 miRNAs showed differential expression (Figure 3 and Additional file 6) including 6 miRNAs in stage 4 larvae (miR-7a, miR-10b, miR-92a, miR-124, miR-130b, and miR-221); 9 miRNAs in stage 8 (pre-metamorphic) larvae (miR-7a, miR-10b, miR-124, miR-130b, miR-192, miR-205, miR-206, miR-218a, and miR-218b); and 4 miRNAs in stage 11 (mid-metamorphosis) larvae (miR-16a, miR-18a, miR-21, and miR-130b).

### Long-term effect of water temperature on miRNA expression levels in juvenile Atlantic cod

**Figure 4:**
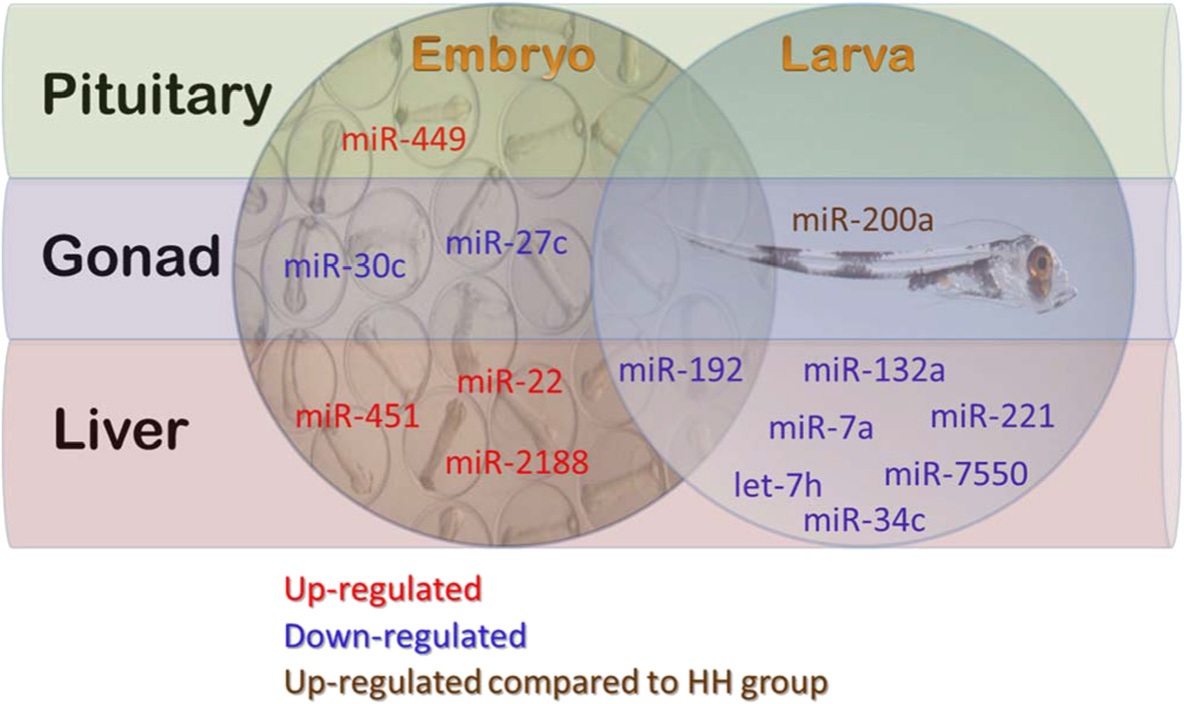
Long-term effects of elevated temperature in early ontogeny on miRNA expression in juvenile Atlantic cod depend on timing. Elevation of temperature during either embryo incubation or larval rearing (see Figure 6) affects different sets of miRNAs, with the exception of miR-192. Differential expression (up- or down-regulation) is given in relation to low temperature group, with the exception of miR-200a, where expression is differential in relation to high temperature group (see Additional file 7).

Sequencing was performed separately for males and females, but no significant differences were found between the sexes within a treatment group. Fourteen miRNAs were differentially expressed among the treatment groups in pituitary, liver, and gonad (Figure 4, Additional files 4 and 7). No significant differential expression was found in brain.

The timing of temperature elevation during early ontogeny was important for differential expression pattern of miRNAs in juvenile Atlantic cod. Here, timing refers to whether the temperature was elevated during embryo incubation (HH group) or later on, during larval rearing (LH group). Except for miR-192, the remaining 13 miRNAs were differentially expressed either in HH or LH groups, but not in both. Interestingly, when compared to the low temperature group, elevated temperature during embryo incubation resulted in up-regulation while elevated temperature during larval rearing resulted in down-regulation of differentially expressed miRNAs in liver (Figure 4).

**Figure 5:**
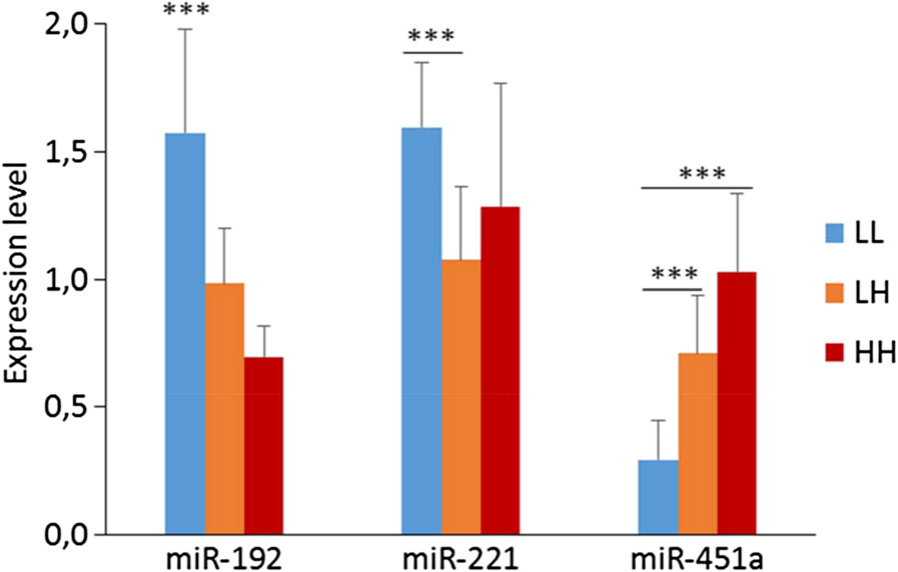
RT-qPCR result for selected miRNAs. Expression value were normalized against miR-122-5p. Vertical bars represent Standard Deviation (n = 7). *** stand for p < 0.001. For the detail descriptions of LL, LH, and HH see Methods.

To test the reliability of NOISeq-simulated technical replicates, we performed quantitative reverse transcription PCR (rt-qPCR) on liver samples of 10 g juveniles from 7 individuals (biological replicates) per each treatment. We observed very similar expression pattern between the NGS analysis (Additional file 7) and the rt-qPCR results for miR-192, miR-221, and miR-451a (Figure 5); however, the amplification of miR-7a was low with Ct values > 40.

### DNA methylation at upstream regions of miRNA genes

We aimed to explore CpG methylation up to 2,000 bp upstream of 21 differentially expressed miRNAs for possible epigenetic footprints. However, due to incompleteness of the sequenced Atlantic cod genome and fragmentary nature of its assembly, our analysis was restricted to 11 upstream regions of pre-miRNAs. In 8 pre-miRNAs upstream regions, there were too few CpG sites to perform methylation pattern analysis, or as in the case of miR-132a where the CpGs were located in the middle of thymidine stretches resulting in sequence ambiguity (data not shown). Thus, the remaining two upstream regions (let-7 h and miR-27c) were amplified in both bisulfite-treated and -untreated DNA by their respective primer pairs. Methylation status of these upstream regions in stage 8 larvae was mostly inconclusive. A notable exception was a single site at −153 bp upstream of pre-miR-27c, which was unmethylated in LL larvae, methylated in HH larvae, and methylated in some larvae in the HL and LH groups (Additional file 8). Then, we analyzed tissue-specific methylation in later developmental stages. The upstream region of let-7h gene in the liver of 10 g juvenile had no differential methylation pattern among the treatment groups and resembled the pattern observed in stage 8 larvae (Additional file 8). We found the methylation pattern of upstream region of miR-27c gene, which was differentially expressed in juvenile gonads (Figure 4 and Additional file 7), did not show any difference between temperature groups in 3-year-old adult gonad (Additional file 8).

## Discussion

### Characterization of miRNA during Atlantic cod early development

We have identified a number of conserved miRNAs, and characterized several species-specific miRNAs, during early development of Atlantic cod. This report provides a systematic characterization of miRNA in this species, which expands the previous mature miRNA profiling in selected developmental stages and liver [23]. Although multiple miRNA loci could be an attribute of fragmentary nature of Atlantic cod genome assembly [24], a majority (77.7%) of our putative precursor miRNAs are found in the scaffolds, indicating that these loci are most likely paralogs, as reported for salmonids [25,26].

The expression pattern of some specific miRNAs in the present study is in agreement with previous studies [23,27,28]. Some miRNAs had high expression level before zygotic genome activation, indicating their maternal origin, as reported in flies [29], mammals [30], and fish [31]. Also, miR-7641 expression was high during the early development, indicating the relevance of this miRNA during cleavages. In mammals, miR-7641 expression is significantly higher in embryonic stem cells compared to differentiated cells [32]. In zebrafish, the maternal-to-zygotic transition (MZT) occurs during mid-blastula stage, but it is not exactly delineated in Atlantic cod. However, similarly to zebrafish [33], peak in miR-430 expression in Atlantic cod was at the blastula stage, suggesting similar MZT window.

In Atlantic cod early development, miR-20 family had the highest expression during somitogenesis, and other miRNAs, such as miR-27b and miR-221 were highly expressed in yolk sac larvae. Previous reports indicate that miR-20 family is essential for normal development; in particular, miR-20a has a function in skeletogenesis [34], and miR-27b and miR-221, identified as proangiogenic miRNA in zebrafish, promote development of intersegmental blood vessels [35-37]. High expression miR-103/107 in Atlantic cod during hatching indicates the importance in the initiation of the digestive tract development as the enrichment of these miRNAs in gut is reported in zebrafish [38]. In the present study, high level of miR-125b and miR-181a in hatched larvae and during further development may be associated with the developmental advancement of different organs as shown in zebrafish and other systems [39-43].

The expression of several miRNAs in Atlantic cod tissues was in agreement with previous reports in homologous tissues of vertebrates. The enrichment of miR-9, miR-124 and miR-128 in a developing and mature central nerve system (CNS) is reported in fish [44], mammals [45], amphibians [46] and birds [47]. Atlantic cod pituitary was enriched with miR-375, which is also highly expressed in mouse intermediate lobe of the pituitary gland [48]. In this study, we found very high expression of miR-202 in gonads, which is in agreement with previous reports in Atlantic halibut [49] and rainbow trout [31], indicating possible functional conservation in developing gonads.

### Effect of temperature on miRNA expression

Although our sampling strategy (see Methods) reduced the discrepancy that could arise from allometric growth of different cell types/tissues, the observed differences in miRNA expression between the two temperature groups should be interpreted cautiously. Some of the differences in miRNA expression could be an effect of heterochrony resulting in slight modification in the respective time of gene activation.

Lower expression of miR-130b and miR-430 in the higher incubation temperature, observed in this study, may partly explain the observed delay of epiboly and closure of the blastopore in Atlantic cod embryos incubated at 10°C compared to 4 or 7°C [50]. miR-130b is involved in promoting cell proliferation [51], which is an important heterochronic process during the epiboly and prior to somite formation. One of the functions of miR-430 is balancing the agonistic (nodal) and antagonistic (lefties) ligands during embryogenesis [52], which are essential for left-right patterning, initiation of germ layers and establishing of distinct cell fates in a dose-dependent manner [53]. miR-430 expression starts after zygotic genome activation, which is triggered, at least in zebrafish, by Nanog, Pou5f1, and SoxB1 transcriptional factors [33]. Previous report showed no significant difference in the expression of *nanog* and *pou2* between Atlantic cod embryos incubated at 6°C and 10°C [54]. Therefore, difference in mR-430 expression between the two temperature groups in the present study may not be at the transcriptional level; alternatively, other transcriptional factors that are involved in the transcription of miR-430 cluster are affected by change in temperature.

Vertebrate miRNAs are involved in regulation of gastrulation and somitogenesis [55-58]. Lowered expression of miR-206 in HH group might have a phenotypic effect, since Atlantic cod embryos incubated at 10°C had shorter body axis than those incubated at 4°C [59]. In zebrafish, balanced concentration of miR-206 is essential for the miR-206/Snail/E-cadherin signaling in the control of convergence and extension movements during gastrulation, which contribute to narrowing and lengthening of the forming embryonic axis [58,60]. Earlier report indicated prevalence of severe vertebral curvature and shortened tails of Atlantic cod larvae occurring after incubation of embryos at above 9°C [61]. However, we did not observe any severe deformities in HH group compared with LH; in contrast, LL group fish had more deformities compared with LL and HH groups at juvenile stage (data not shown). In zebrafish, miR-19 family, a member of miR-17 ~ 92 cluster, regulates both retinoic acid metabolism during early somitogenesis and axis formation [62]. In this study, members of miR-17 ~ 92: miR-17, miR-18c, miR-19b, and miR-20a, had lower expression in HH group during early somites stage, indicating the effect of temperature on somitogenesis. In Senegalese sole (*Solea senegalensis*) at 20 somites stage, higher expression of miR-130c and miR-181a-3p was reported in embryos incubated at lower temperature (15°C) compared with higher temperature (21°C) [21]. Thus, the observed differences between LL and HH groups in this study suggest the possible temperature-dependent modulation of miRNA expression.

Transfer of larvae from low to high temperature (LL to LH) changed the expression profile of 6 miRNAs. These miRNAs are implied in neuronal development, endocrine development, adipogenesis, angiogenesis and bone formation (Additional file 9). Among these 6 miRNAs, only miR-7 has been implicated in stabilization of gene expression to temperature fluctuation in flies [63]. However, role of these miRNAs in relation to temperature is not yet explored in vertebrates.

Temperature is known to have an influence on the size and number of embryonic muscle fibers [64]. Hall and Johnston [50] have reported significant change in muscle cellularity with more white fibers in Atlantic cod embryos incubated at 10°C compared to those incubated at lower temperature. In this study, lower temperature (4°C) promoted miR-206 expression during larval development, which may imply the role of miR-206 in muscle cellularity.

### Long-term effect of incubation temperature during embryonic and larval stages on miRNA expression in juveniles

Incubation temperature experienced in early life stages showed long-term effects on the expression of some miRNAs in pituitary, gonads and liver, but not in brain of juveniles (Additional file 7). The lack of incubation temperature effects in brain is in agreement with a report that has shown unaltered miRNAs expression in the brain tissue of cold-acclimatized zebrafish [22]. However, given that brain has several anatomical and functionally distinct parts, this result is inconclusive.

Long-term effects of temperature were time-dependent, as different sets of miRNAs were differentially expressed in the HH group (temperature altered during embryo incubation) and in the LH group (temperature altered later on, during larval rearing; Figure 4). It indicates that a time-specific window predominantly exists for temperature-induced long-term changes in miRNA expression during Atlantic cod development. Intriguingly, only three out of the 14 differentially expressed miRNAs in 10 g fish (miR-192, miR-221 and miR-7a) were among miRNAs differentially expressed during either embryonic or larval stages (Figure 3). It indicates that temperature-induced response in altered miRNA expression is mostly prolonged in time, and it suggests an epigenetic mechanism. Our effort to identify DNA methylation-based regulatory network for miRNAs was mostly inconclusive, owing to lack of good Atlantic cod genome assembly and thereby limiting the number of miRNAs examined. However, we found apparent differences in methylation status at −153 bp of miR-27c gene in stage 8 larvae, unmethylated in LL group and methylated in HH group (Additional file 8) and this corresponds well with highly significant down-regulation in miR-27c expression in a juvenile gonad in HH group (Additional file 7). Using transcription factor binding site database [65], we predicted transcriptional factor GATA1 to bind at positions −146 to −154 bp (AATATCGCG) up-stream of miR-27c gene. This may indicate epigenetic modulation of miR-27c expression by temperature. However, more experimental evidence is needed to support a hypothesis on epigenetic modulation of temperature-induced differential miRNA expression. miR-192 was the only exception from the time-specific window for temperature-dependent modulation of miRNA expression, as it was affected in both HH and LH groups (Figure 4). Moreover, it was altered directly during the treatment at stage 8 larvae (Figure 3). miR-192 is involved in immune response [66,67] and since temperature is a determinant in Atlantic cod immune response [68], the response to temperature likely follows wide time-sensitive window during the development.

The elevation of temperature during larval development influenced the expression of let-7 h, miR-7a, miR-34c, miR-132a, miR-221 and miR-7550 in the liver of a juvenile Atlantic cod, in all cases producing down-regulation in comparison to LL group. The functions of these miRNAs in fish liver during the development, as well as functions in temperature response are unknown.

Elevation of embryonic incubation temperature resulted in alteration of expression of several miRNAs in juvenile organs. Three miRNAs were up-regulated in the liver (Figure 4), including miR-22, which is involved in metabolic pathways in rat [69]; miR-451, which promotes erythroid maturation in zebrafish [70]; and miR-2188, which is implicated in embryonic angiogenesis in zebrafish [71]. However, their functions in the liver are unknown. The two miRNAs, miR-27c and miR-30c, which were expressed differentially in gonads, have not been implicated in gonad development yet. In human pituitary, miR-449a plays an important role in stress response [72]. Temperature elevation could produce stress response and in consequence long-term alteration of miR-449a expression in pituitary in HH group fish. Embryonic thermal experience affects later growth performance in Atlantic salmon (*Salmo salar*) [73]. Thus, the observed difference in expression of several miRNAs in the present study might be related to metabolic pathways and are likely to influence the growth performance of Atlantic cod. In addition, many of these miRNAs are also involved in immune-related pathways (Additional file 9).

## Conclusions

We provided a systematic identification and characterization of miRNAs during Atlantic cod development in order to elucidate the effect of temperature during early life stages on miRNA expression. We found that several miRNAs show temperature-dependent differential expression during the development. Moreover, we found that temperature during early life stages had long-term effects manifested in differential expression of a number of miRNAs in juveniles, and these effects were inducible predominantly in a narrow time-specific window during the development. Our results indicate that thermal experience during early ontogeny has long-lasting consequences in regulation of biological processes in Atlantic cod, but it is unclear whether this effect is modulated by an epigenetic mechanism.

## Methods

### Animal rearing and experimental setup

The experiment was conducted at National Cod Breeding Station (Kraknes, Tromsø, Norway). All working procedures complied with the Norwegian Regulation on Animal Experimentation (The Norwegian Animal Protection Act, No. 73 of 20 December 1974) and were approved by the National Animal Research Authority (Utvalg for forsøk med dyr, forsøksdyrutvalget, Norway) General License for Fish Maintenance and Breeding (Godkjenning av avdeling for forsøksdyr) no. 17.

**Figure 6:**
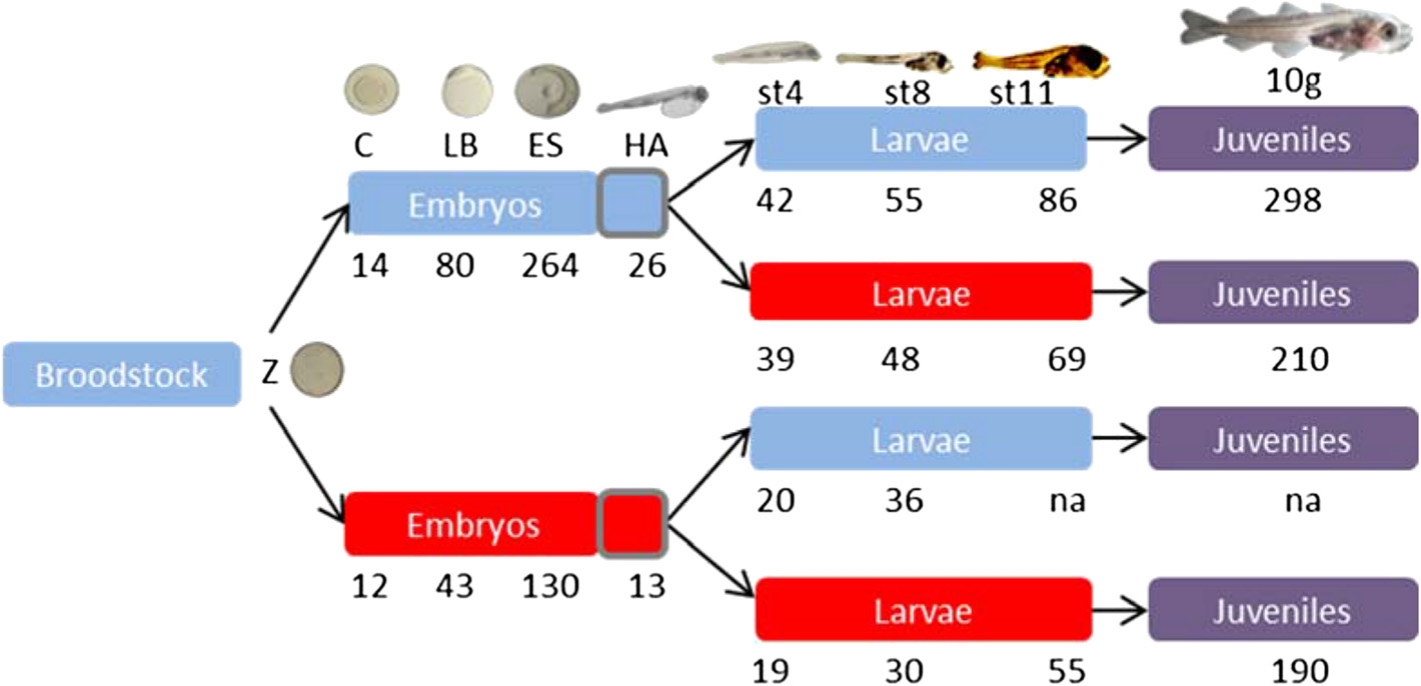
Experimental setup. Larval developmental staging was according to Herbing *et al*. [75]. Z, C, LB, ES, HA, st4, st8, and st11 stand for zygote, cleavage, late blastula, early somitogenesis, hatch, stage 4 larvae, stage 8 larvae, and stage 11 larvae, respectively; 10 g stands for tissues (brain, pituitary, liver, and gonad) from 10 g juveniles. Blue, red, and purple boxes represent 4°C, 9.5°C, and 7°C incubation temperatures, respectively. The numbers under each treatment groups show age of embryos (hours post fertilization), hatchlings, larvae, and juveniles (days post fertilization) at a given sampling point. Note that sampling days differ among the groups because of temperature-dependent developmental pace. “na” denotes no samples taken (not available) because of low survival in HL group. The detailed description of LL, LH, HL, and HH is given in the text.

Brood fish that originated from the second generation from the National Cod Breeding Program were kept at 4–5°C and gametes were collected by hand stripping. After fertilization, eggs were transferred to 16 incubation tanks, from which 8 tanks were maintained at low temperature (4 ± 0.5°C), whereas the remaining tanks were maintained at high temperature (9.5 ± 0.5°C), which was reached by gradual increment (~0.5°C every 3 h) [74]. Hatched larvae were transferred to first feeding tanks (200 L circular tanks) and the temperature was kept either at 4 or 9.5°C at transfer. From 5 days post hatch, temperature in 4 tanks from low temperature incubation group (4°C) was gradually increased to 9.5°C at ~0.9°C d^−1^ (LH), while the temperature in the remaining 4 tanks was continuously kept at 4°C (LL). Likewise, from 5 days post hatch temperature in 4 tanks from high temperature group (9.5°C) was gradually decreased to 4°C (HL), while the temperature in the other 4 tanks was kept at 9.5°C (HH). Juveniles from all treatment groups were maintained at 7°C (Figure 6).

### Sampling and RNA extraction

Larval developmental staging was performed according to Herbing *et al*. [75]. Sampling was performed based on developmental advancement rather than age, because of differences in the pace of cod development among the treatments (Figure 6). Embryos at zygote, cleavage, late blastula, early somitogenesis and hatch, as well as larvae at stage 4, stage 8 and stage 11 were collected. Brain, pituitary, liver, and gonads were collected from 10 g juveniles, and gonads were sampled from 3-year-old adults. Sexing of 10 g juveniles was performed using gonadal aromatase (*cyp19a*) and Anti-Müllerian hormone (*Amh*) gene expression [76]. Samples were snap-frozen in liquid nitrogen and stored at −80°C until processed. RNA was extracted from ~100 mg (embryos, larvae, or tissues) from 3 replicates using TRIzol reagent (Invitrogen, Carlsbad, California, USA). The quantity and quality of total RNA were examined using the Agilent 2100 bioanalyzer (Agilent technologies, Waldbronn, Germany), and samples with RNA integrity number greater than 7 were used for sequencing. RNA samples from developmental stage 4 in HL and HH groups, as well as stage 11 and juveniles from HL group were not analyzed because of poor RNA quality and/or poor survival.

### Sequencing and sequence analysis

Equal amount of total RNA from the replicates were pooled to prepare sequencing libraries. For 10 g juvenile tissues, libraries from males and females were prepared separately. Libraries were prepared following SOLiD Whole Trascriptome Analysis Kit protocol (Life Technologies, Austin, USA). Sequencing was performed on 6 lanes of a slide using 5500xl SOLiD sequencer (Life Technologies, Tokyo, Japan) at the University of Nordland, Bodø, Norway. After removing low quality (quality score < 20) and less complex sequences, adapter sequences were removed using cutadapt [77] and the trimmed sequences were mapped to Atlantic cod genome (gadMor1, Ensembl, release 73) using Bowtie [78]. Hairpin structures were predicted using miRDeep2 [79]. The identified hairpins were further evaluated and redundant sequences were removed. To identify conserved precursor miRNAs, the sequences were blasted against miRBase Version20 (http://www.mirbase.org/). To remove other known non-coding RNAs, the remaining sequences were blasted against Rfam 11.0. (http://rfam.sanger.ac.uk/). The sequencing data is submitted to miRBase database (http://www.mirbase.org/).

Read counts were normalized using DESeq method, which assumes most genes are not differentially expressed [80]. The scaling factor is estimated by taking the median of the ratio of a sample read counts divided by the geometric mean across all samples [80]. This normalization method is robust in controlling false positive rate, intra-variance, and has better power in the presence of different library sizes [81]. miRNAs with low count were filtered using CPM method (cutoff = 100 and cpm = 10), and the expression levels of mature miRNAs were assessed using NOISeq [82]. Since sequencing was done without biological replicates, differential expression probability was estimated using simulated 5 technical replicates. The simulation was performed by replacing all zero counts by 0.5 (*k* = 0.5), each simulated replicate had 20 % of the total reads of a given sample (pnr = 0.2) and 2 % variability in the size of each simulated replicate (*v* =0.02) [82]. Given that the technical replicates are an approximation, we set stringent NOISeq parameters to identify expression changes between treatments; i.e., high probability of differential expression (q > 0.99) and high normalized sequence counts difference between groups under comparison (>10,000). The second consideration is important in case of low sequence counts, in which fold change may overestimate the difference in miRNA expression between groups.

We performed hierarchical clustering and heatmap of miRNA families after logarithm transformation of normalized reads using heatmap2 in R (http://www.r-project.org/).

### RT-qPCR

The reliability of NOISeq technical replicates was tested using LNA-based rt-qPCR for 10 g juvenile liver. We used 7 individuals for each treatment to examine the expression level of miR-7a, miR-122, miR-192, miR-221, and mir-451 (Additional file 10) using miRCURY LNA Universal RT microRNA PCR (Exiqon, Vedbaek, Denmark). The reaction was performed on Light Cycler 480 (Roche Applied Science, Rotkreuz, Switzerland) using white 96 well plates with thermal cycle conditions of 95°C for 10 min, followed by 45 cycles of 95°C for 10 s and 60°C for 1 min. Cq values were collected using 2^nd^ derivative method. The relative expression of each miRNA in a sample was normalized against miR-122 expression, which was stably expressed among treatments. Statistical differences were computed with one-way ANOVA, followed by Tukey's HSD multiple comparison *post-hoc* test.

### Methylation analysis

Genomic DNA was extracted from stage 8 larvae (n = 7 per each temperature group), 10 g juvenile liver (n = 7), and 3-year-old adult gonad (n = 14) using MasterPure-complete DNA and RNA purification kit (Epicenter, Madison, Wisconsin, USA) following the manufacturer procedure. DNA samples were treated with sodium bisulfite using the EZ DNA Methylation-Gold kit (Zymo Research, Irvine, California, USA) following the manufacturer protocol. For signatures of CpG sites methylation, 5′ upstream regions of pre-miRNAs (−2000 to 0 bp) were examined. Then, we designed two sets of primers for bisulfite-treated and -untreated DNA sequences (Additional file 10), which flanked CpG dinucleotides. Methylation status was analyzed by direct Sanger sequencing of the PCR products.

## Availability of supporting data

The data generated in this study are given in Additional Files and submitted to miRBase (www.mirbase.org).

## Additional files

Additional file 1:
**Size distribution of sequences obtained from small RNA sequencing of Atlantic cod early developmental stages and four juvenile tissues.**
Additional file 2:
**Sequences of conserved and novel precursor miRNAs and mature miRNAs and their genomic location in the assembled Atlantic cod genome (gadMor1, Ensembl, release 73).**
Additional file 3:
**Heatmap of miRNA expression during Atlantic cod embryonic and larval development (8 developmental stages) at 4°C.** High and low expression is marked with red and green, respectively. Additional file 4:
**Heatmap of miRNA expression in 4 organs and tissues of 10 g juvenile Atlantic cod.** Fish were reared under 3 temperature regimes (LL, LH and HH). For details of the temperature regimes see Methods. B, P, G, and L stand for brain, pituitary, gonad, and liver, respectively. High and low expression is marked with red and green, respectively. Additional file 5:
**Mature miRNA counts in various developmental stages and tissues of Atlantic cod incubated/reared under different temperature regimes.** F and M stand for female and male, respectively. LL, LH, and HH stand for temperature regimes (for details see Methods). Additional file 6:
**Differential expression of miRNAs during Atlantic cod embryonic and larval development under four temperature regimes, based on pair-wise comparison between treatments.** * indicates significant q-value, which is the probability of differential expression, > 0.99. LL, LH, HL, and HH are temperature regimes described in Methods. C, BL, ES, HA, St4, St8, and St11 stand for cleavage, blastula, early somitogenesis, hatch, stage 4 larvae, stage 8 larvae, and stage 11 larvae developmental stages, respectively. Additional file 7:
**Differential expression of miRNAs in pituitary, gonads, and liver of Atlantic cod juveniles reared in three temperature regimes.** * stands for significant q-value, which is the probability of differential expression > 0.99. LL, LH, and HH are temperature regimes described in Methods. Additional file 8:
**DNA methylation status at upstream region of miRNA genes in stage 8 larvae, 10 g juvenile liver, and in gonads of adult individuals.** The percentage of individuals with methylated (blue) and unmethylated (black) DNA at specific CpG position is displayed. CpG positions are labeled from the start of the pre-miRNA with negative numbers. Numbers of individuals examined in each temperature group are given in brackets. LL, LH, HL, and HH are temperature regimes described in Methods. Additional file 9:
**Differentially expressed miRNAs during Atlantic cod development and some of the functions of their homologs in other species.**
Additional file 10:
**Primers.** Top table: primers used to amplify the bisulfite treated and untreated DNA. Bottom table: LNA primers used for rt-qPCR and their efficiency.

## Abbreviations

IPCC: Intergovernmental Panel on Climate Change
HH: Embryos and larvae were kept continuously at high temperature (9.5°C)
HL: Embryos were incubated at high temperature (9.5°C) and then larvae were reared at low temperature (4°C)
LH: Embryos were incubated at low temperature (4°C) and then larvae were reared at high temperature (9.5°C)
LL: Embryos and larvae were kept continuously at low temperature (4°C)
MZT: Maternal to Zygotic Transition
POMC: Pro-opiomelanocortin
SOLiD: Sequencing by Oligo Ligation and Detection

## References

[1] IPCC. Climate Change 2013: The Physical Science Basis. Contribution of Working Group I to the Fifth Assessment Report of the Intergovernmental Panel on Climate Change. In. Edited by Stocker TF, Qin D, Plattner G-K, Tignor M, Allen SK, Boschung J, Nauels A, Xia Y, Bex V, Midgley PM. Cambridge University Press, Cambridge, U. K. 2013

[2] Drinkwater KF (2005). The response of Atlantic cod (Gadus morhua) to future climate change. ICES J Marine Sci.

[3] Hollowed AB, Barange M, Beamish RJ, Brander K, Cochrane K, Drinkwater K, Foreman MGG, Hare JA, Holt J, Ito SI (2013). Projected impacts of climate change on marine fish and fisheries. ICES J Marine Sci.

[4] Petitgas P, Rijnsdorp AD, Dickey-Collas M, Engelhard GH, Peck MA, Pinnegar JK, Drinkwater K, Huret M, Nash RDM (2013). Impacts of climate change on the complex life cycles of fish. Fish Oceanogr.

[5] Pörtner HO, Berdal B, Blust R, Brix O, Colosimo A, De Wachter B, Giuliani A, Johansen T, Fischer T, Knust R (2001). Climate induced temperature effects on growth performance, fecundity and recruitment in marine fish: developing a hypothesis for cause and effect relationships in Atlantic cod (Gadus morhua) and common eelpout (Zoarces viviparus). Cont Shelf Res.

[6] Stenevik EK, Sundby S (2007). Impacts of climate change on commercial fish stocks in Norwegian waters. Mar Policy.

[7] Stensholt BK (2001). Cod migration patterns in relation to temperature: analysis of storage tag data. ICES J Marine Sci.

[8] Perry AL, Low PJ, Ellis JR, Reynolds JD (2005). Climate change and distribution shifts in marine fishes. Science.

[9] Pepin P, Orr DC, Anderson JT (1997). Time to hatch and larval size in relation to temperature and egg size in Atlantic cod (Gadus morhua). Can J Fish Aquat Sci.

[10] Pörtner HO, Farrell AP (2008). Physiology and climate change. Science.

[11] O'Brien CM, Fox CJ, Planque B, Casey J (2000). Fisheries: Climate variability and North Sea cod. Nature.

[12] Le Bris A, Fréchet A, Galbraith PS, Wroblewski JS (2013). Evidence for alternative migratory behaviours in the northern Gulf of St Lawrence population of Atlantic cod (Gadus morhua L.). ICES J Marine Sci.

[13] Tattersall GJ, Sinclair BJ, Withers PC, Fields PA, Seebacher F, Cooper CE, Maloney SK (2012). Coping with thermal challenges: Physiological adaptations to environmental temperatures. Comprehensive Physiol.

[14] Somero GN (2010). The physiology of climate change: how potentials for acclimatization and genetic adaptation will determine ‘winners’ and ‘losers’. J Exp Biol.

[15] Pasquinelli AE (2012). MicroRNAs and their targets: recognition, regulation and an emerging reciprocal relationship. Nat Rev Genet.

[16] Bizuayehu TT, Babiak I (2014). MicroRNA in Teleost Fish. Genome Biol Evol.

[17] Garcia De La Serrana D, Vieira VLA, Andree KB, Darias M, Estévez A, Gisbert E, Johnston IA (2012). Development Temperature Has Persistent Effects on Muscle Growth Responses in Gilthead Sea Bream. PLoS One.

[18] Johnston IA, Lee HT, Macqueen DJ, Paranthaman K, Kawashima C, Anwar A, Kinghorn JR, Dalmay T (2009). Embryonic temperature affects muscle fibre recruitment in adult zebrafish: genome-wide changes in gene and microRNA expression associated with the transition from hyperplastic to hypertrophic growth phenotypes. J Exp Biol.

[19] Scott GR, Johnston IA (2012). Temperature during embryonic development has persistent effects on thermal acclimation capacity in zebrafish. Proc Natl Acad Sci.

[20] Jonsson B, Jonsson N (2014). Early environment influences later performance in fishes. J Fish Biol.

[21] Campos C, Sundaram AYM, Valente LMP, Conceicao LEC, Engrola S, Fernandes JMO (2014). Thermal plasticity of the miRNA transcriptome during Senegalese sole development. BMC Genomics.

[22] Yang R, Dai Z, Chen S, Chen L (2011). MicroRNA-mediated gene regulation plays a minor role in the transcriptomic plasticity of cold-acclimated zebrafish brain tissue. BMC Genomics.

[23] Johansen SD, Karlsen BO, Furmanek T, Andreassen M, Jorgensen TE, Bizuayehu TT, Breines R, Emblem A, Kettunen P, Luukko K (2011). RNA deep sequencing of the Atlantic cod transcriptome. Comp Biochem Physiol D Genomics Proteomics.

[24] Star B, Nederbragt AJ, Jentoft S, Grimholt U, Malmstrom M, Gregers TF, Rounge TB, Paulsen J, Solbakken MH, Sharma A (2011). The genome sequence of Atlantic cod reveals a unique immune system. Nature.

[25] Berthelot C, Brunet F, Chalopin D, Juanchich A, Bernard M, Noël B, Bento P, Da Silva C, Labadie K, Alberti A (2014). The rainbow trout genome provides novel insights into evolution after whole-genome duplication in vertebrates. Nat Commun.

[26] Bekaert M, Lowe NR, Bishop SC, Bron JE, Taggart JB, Houston RD (2013). Sequencing and characterisation of an extensive Atlantic salmon (*Salmo salar* L.) microRNA repertoire. PLoS One.

[27] Bizuayehu T, Lanes C, Furmanek T, Karlsen B, Fernandes J, Johansen S, Babiak I (2012). Differential expression patterns of conserved miRNAs and isomiRs during Atlantic halibut development. BMC Genomics.

[28] Giraldez AJ, Mishima Y, Rihel J, Grocock RJ, Van Dongen S, Inoue K, Enright AJ, Schier AF (2006). Zebrafish miR-430 promotes deadenylation and clearance of maternal mRNAs. Science.

[29] Soni K, Choudhary A, Patowary A, Singh AR, Bhatia S, Sivasubbu S, Chandrasekaran S, Pillai B (2013). MiR-34 is maternally inherited in *Drosophila melanogaster* and *Danio rerio*. Nucleic Acids Res.

[30] Tang F, Kaneda M, O’Carroll D, Hajkova P, Barton SC, Sun YA, Lee C, Tarakhovsky A, Lao K, Surani MA (2007). Maternal microRNAs are essential for mouse zygotic development. Genes Dev.

[31] Ma H, Hostuttler M, Wei H, Rexroad CE, Yao J (2012). Characterization of the rainbow trout egg microRNA transcriptome. PLoS One.

[32] Yoo J, Jung H, Kim C-H, Son W, Kim J (2013). miR-7641 modulates the expression of CXCL1 during endothelial differentiation derived from human embryonic stem cells. Arch Pharmacal Res.

[33] Lee MT, Bonneau AR, Takacs CM, Bazzini AA, DiVito KR, Fleming ES, Giraldez AJ (2013). Nanog, Pou5f1 and SoxB1 activate zygotic gene expression during the maternal-to-zygotic transition. Nature.

[34] Tiago DM, Marques CL, Roberto VP, Cancela ML, Laizé V (2014). Mir-20a regulates in vitro mineralization and BMP signaling pathway by targeting BMP-2 transcript in fish. Arch Biochem Biophys.

[35] Nicoli S, Knyphausen C-P, Zhu Lihua J, Lakshmanan A, Lawson Nathan D (2012). miR-221 is required for endothelial tip cell behaviors during vascular development. Dev Cell.

[36] Biyashev D, Veliceasa D, Topczewski J, Topczewska JM, Mizgirev I, Vinokour E, Reddi AL, Licht JD, Revskoy SY, Volpert OV (2012). miR-27b controls venous specification and tip cell fate. Blood.

[37] Concepcion CP, Bonetti C, Ventura A (2012). The microRNA-17-92 family of microRNA clusters in development and disease. Cancer J.

[38] Soares A, Pereira P, Santos B, Egas C, Gomes A, Arrais J, Oliveira J, Moura G, Santos M (2009). Parallel DNA pyrosequencing unveils new zebrafish microRNAs. BMC Genomics.

[39] Le MTN, Shyh-Chang N, Khaw SL, Chin LZ, Teh C, Tay J, O'Day E, Korzh V, Yang H, Lal A (2011). Conserved regulation of p53 network dosage by microRNA-125b occurs through evolving miRNA-target gene pairs. PLoS Genet.

[40] Le MTN, Teh C, Shyh-Chang N, Xie H, Zhou B, Korzh V, Lodish HF, Lim B (2009). MicroRNA-125b is a novel negative regulator of p53. Genes Dev.

[41] Dunworth WP, Cardona-Costa J, Cagavi E, Kim J-d, Fischer JC, Meadows S, Wang Y, Cleaver O, Qyang Y, Ober EA (2013). Bone morphogenetic protein 2 signaling negatively modulates lymphatic development in vertebrate embryos. Circul Res.

[42] Bhushan R, Grünhagen J, Becker J, Robinson PN, Ott C-E, Knaus P (2013). miR-181a promotes osteoblastic differentiation through repression of TGF-β signaling molecules. Int J Biochem Cell Biol.

[43] Li Q-J, Chau J, Ebert PJR, Sylvester G, Min H, Liu G, Braich R, Manoharan M, Soutschek J, Skare P (2007). miR-181a is an intrinsic modulator of T cell sensitivity and selection. Cell.

[44] Kapsimali M, Kloosterman WP, de Bruijn E, Rosa F, Plasterk RHA, Wilson SW (2007). MicroRNAs show a wide diversity of expression profiles in the developing and mature central nervous system. Genome Biol.

[45] Smirnova L, Grafe A, Seiler A, Schumacher S, Nitsch R, Wulczyn FG (2005). Regulation of miRNA expression during neural cell specification. Eur J Neurosci.

[46] Yu J-Y, Chung K-H, Deo M, Thompson RC, Turner DL (2008). MicroRNA miR-124 regulates neurite outgrowth during neuronal differentiation. Exp Cell Res.

[47] Visvanathan J, Lee S, Lee B, Lee JW, Lee S-K (2007). The microRNA miR-124 antagonizes the anti-neural REST/SCP1 pathway during embryonic CNS development. Genes Dev.

[48] Zhang N, Lin JK, Chen J, Liu XF, Liu JL, Luo HS, Li YQ, Cui S (2013). MicroRNA 375 Mediates the Signaling Pathway of Corticotropin-releasing Factor (CRF) Regulating Pro-opiomelanocortin (POMC) Expression by Targeting Mitogen-activated Protein Kinase 8. J Biol Chem.

[49] Bizuayehu TT, Babiak J, Norberg B, Fernandes JMO, Johansen SD, Babiak I (2012). Sex-Biased miRNA Expression in Atlantic Halibut (*Hippoglossus hippoglossus*) Brain and Gonads. Sex Dev.

[50] Hall T, Johnston I (2003). Temperature and developmental plasticity during embryogenesis in the Atlantic cod *Gadus morhua* L. Mar Biol.

[51] Yeung ML, Yasunaga J-i, Bennasser Y, Dusetti N, Harris D, Ahmad N, Matsuoka M, Jeang K-T (2008). Roles for microRNAs, miR-93 and miR-130b, and tumor protein 53–induced nuclear protein 1 tumor suppressor in cell growth dysregulation by human T-cell lymphotrophic virus 1. Cancer Res.

[52] Choi W-Y, Giraldez AJ, Schier AF (2007). Target protectors reveal dampening and balancing of nodal agonist and antagonist by miR-430. Science.

[53] Shen MM (2007). Nodal signaling: developmental roles and regulation. Development.

[54] Skjærven KH, Olsvik PA, Finn RN, Holen E, Hamre K (2011). Ontogenetic expression of maternal and zygotic genes in Atlantic cod embryos under ambient and thermally stressed conditions. Comparative Biochem Physiol A.

[55] Giraldez AJ, Cinalli RM, Glasner ME, Enright AJ, Thomson JM, Baskerville S, Hammond SM, Bartel DP, Schier AF (2005). MicroRNAs regulate brain morphogenesis in zebrafish. Science.

[56] Rathjen T, Pais H, Sweetman D, Moulton V, Munsterberg A, Dalmay T (2009). High throughput sequencing of microRNAs in chicken somites. FEBS Lett.

[57] Goljanek-Whysall K, Sweetman D, Abu-Elmagd M, Chapnik E, Dalmay T, Hornstein E, Münsterberg A (2011). MicroRNA regulation of the paired-box transcription factor Pax3 confers robustness to developmental timing of myogenesis. Proc Natl Acad Sci U S A.

[58] Liu X, Ma Y, Zhang C, Wei S, Cao Y, Wang Q (2013). Nodal promotes mir-206 expression to control convergence and extension movements during zebrafish gastrulation. J Genet Genomics.

[59] Hall TE, Cole NJ, Johnston IA (2003). Temperature and the expression of seven muscle-specific protein genes during embryogenesis in the Atlantic cod Gadus morhua L. J Exp Biol.

[60] Liu X, Ning G, Meng A, Wang Q (2012). MicroRNA-206 regulates cell movements during zebrafish gastrulation by targeting prickle1a and regulating c-Jun N-terminal kinase 2 phosphorylation. Mol Cell Biol.

[61] Fitzsimmons SD, Perutz M (2006). Effects of egg incubation temperature on survival, prevalence and types of malformations in vertebral column of Atlantic cod (Gadus morhua) larvae. Bull Eur Assoc Fish Pathol.

[62] Franzosa JA, Bugel SM, Tal TL, La Du JK, Tilton SC, Waters KM, Tanguay RL (2013). Retinoic acid-dependent regulation of miR-19 expression elicits vertebrate axis defects. FASEB J.

[63] Li X, Cassidy JJ, Reinke CA, Fischboeck S, Carthew RW (2009). A microRNA imparts robustness against environmental fluctuation during development. Cell.

[64] Johnston IA, Bower NI, Macqueen DJ (2011). Growth and the regulation of myotomal muscle mass in teleost fish. J Exp Biol.

[65] Heinemeyer T, Wingender E, Reuter I, Hermjakob H, Kel AE, Kel OV, Ignatieva EV, Ananko EA, Podkolodnaya OA, Kolpakov FA (1998). Databases on transcriptional regulation: TRANSFAC, TRRD and COMPEL. Nucleic Acids Res.

[66] Xia JH, He XP, Bai ZY, Yue GH (2011). Identification and Characterization of 63 MicroRNAs in the Asian Seabass *Lates calcarifer*. PLoS One.

[67] Wu TH, Pan CY, Lin MC, Hsieh JC, Hui CF, Chen JY (2012). In vivo screening of zebrafish microRNA responses to bacterial infection and their possible roles in regulating immune response genes after lipopolysaccharide stimulation. Fish Physiol Biochem.

[68] Perez-Casanova JC, Rise ML, Dixon B, Afonso LO, Hall JR, Johnson SC, Gamperl AK (2008). The immune and stress responses of Atlantic cod to long-term increases in water temperature. Fish Shellfish Immunol.

[69] Koturbash I, Melnyk S, James SJ, Beland FA, Pogribny IP (2013). Role of epigenetic and miR-22 and miR-29b alterations in the downregulation of Mat1a and Mthfr genes in early preneoplastic livers in rats induced by 2-acetylaminofluorene. Mol Carcinog.

[70] Pase L, Layton JE, Kloosterman WP, Carradice D, Waterhouse PM, Lieschke GJ (2009). miR-451 regulates zebrafish erythroid maturation in vivo via its target gata2. Blood.

[71] Soares AR, Reverendo M, Pereira PM, Nivelles O, Pendeville H, Bezerra AR, Moura GR, Struman I, Santos MAS (2012). Dre-miR-2188 targets Nrp2a and mediates proper intersegmental vessel development in zebrafish embryos. PLoS One.

[72] Nemoto T, Mano A, Shibasaki T (2013). miR-449a contributes to glucocorticoid-induced CRF-R1 downregulation in the pituitary during stress. Mol Endocrinol.

[73] Finstad AG, Jonsson B (2012). Effect of incubation temperature on growth performance in Atlantic salmon. Mar Ecol Prog Ser.

[74] Puvanendran V, Falk-Petersen I-B, Lysne H, Tveiten H, Toften H, Peruzzi S. Effects of different step-wise temperature increment regimes during egg incubation of Atlantic cod (Gadus morhua L.) on egg viability and newly hatched larval quality. Aquacult Res 2013:doi: 10.1111/are.12173.

[75] Herbing IHV, Miyake T, Hall BK, Boutilier RG (1996). Ontogeny of feeding and respiration in larval Atlantic cod Gadus morhua (Teleostei, Gadiformes): I Morphology. J Morphol.

[76] Haugen T, Almeida F, Andersson E, Bogerd J, Male R, Skaar K, Schulz R, Sorhus E, Wijgerde T, Taranger G (2012). Sex differentiation in Atlantic cod (Gadus morhua L.): morphological and gene expression studies. Reprod Biol Endocrinol.

[77] Martin M (2011). Cutadapt removes adapter sequences from high-throughput sequencing reads. EMBnet J.

[78] Langmead B, Trapnell C, Pop M, Salzberg S (2009). Ultrafast and memory-efficient alignment of short DNA sequences to the human genome. Genome Biol.

[79] Friedländer MR, Mackowiak SD, Li N, Chen W, Rajewsky N (2012). miRDeep2 accurately identifies known and hundreds of novel microRNA genes in seven animal clades. Nucleic Acids Res.

[80] Anders S, Huber W (2010). Differential expression analysis for sequence count data. Genome Biol.

[81] Dillies M-A, Rau A, Aubert J, Hennequet-Antier C, Jeanmougin M, Servant N, Keime C, Marot G, Castel D, Estelle J (2012). A comprehensive evaluation of normalization methods for Illumina high-throughput RNA sequencing data analysis. Brief Bioinform.

[82] Tarazona S, García-Alcalde F, Dopazo J, Ferrer A, Conesa A (2011). Differential expression in RNA-seq: A matter of depth. Genome Res.

