# Supplementary figures and images for: Temperature during early development has long-term effects on microRNA expression in Atlantic cod
Source: BMC Genomics. 2015 Apr 17;16(1):305. doi: 10.1186/s12864-015-1503-7 (PMC4403832; doi:10.1186/s12864-015-1503-7)

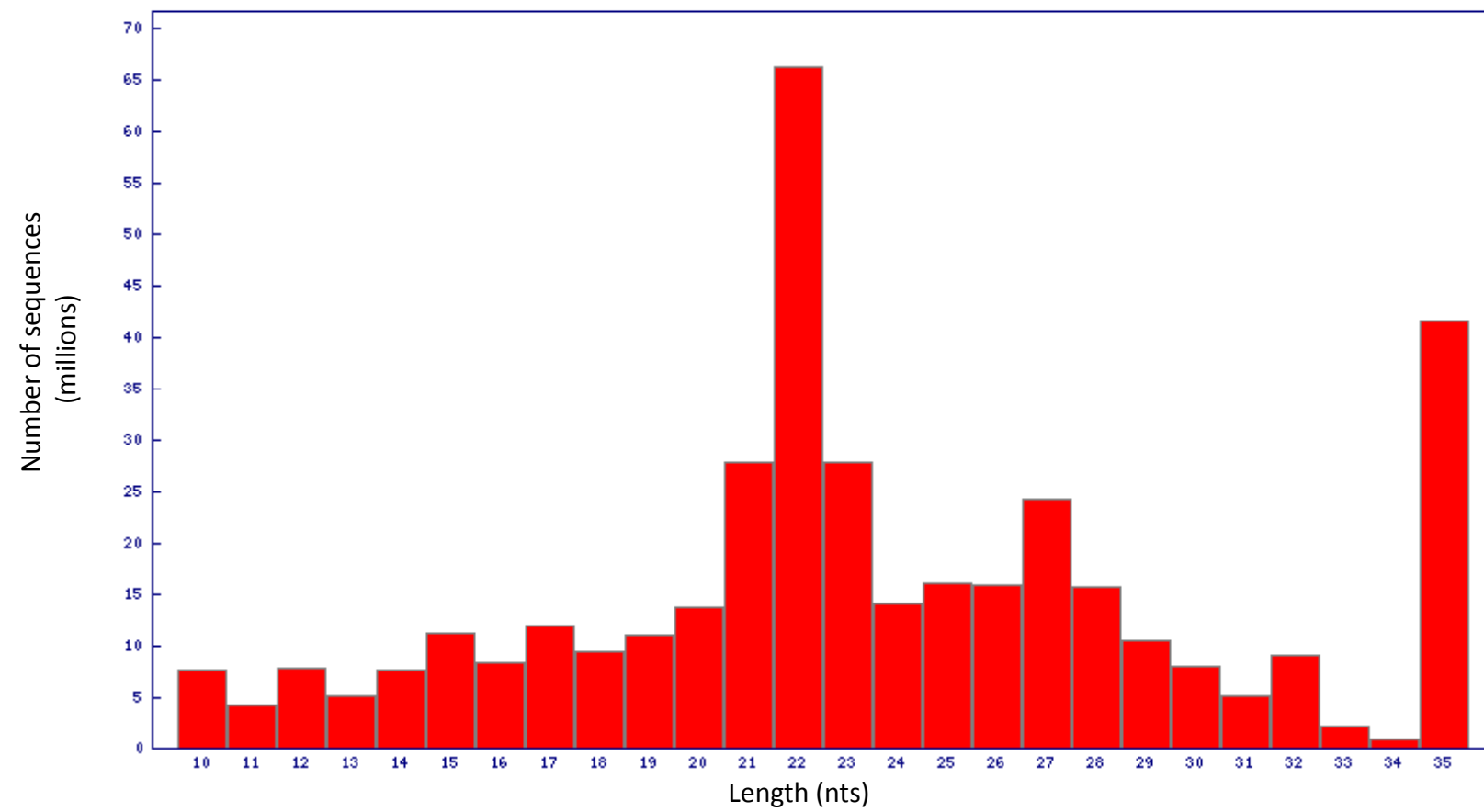

Supplement: Additional file 1: — Size distribution of sequences obtained from small RNA sequencing of Atlantic cod early developmental stages and four juvenile tissues. [file 12864_2015_1503_MOESM1_ESM.pdf]

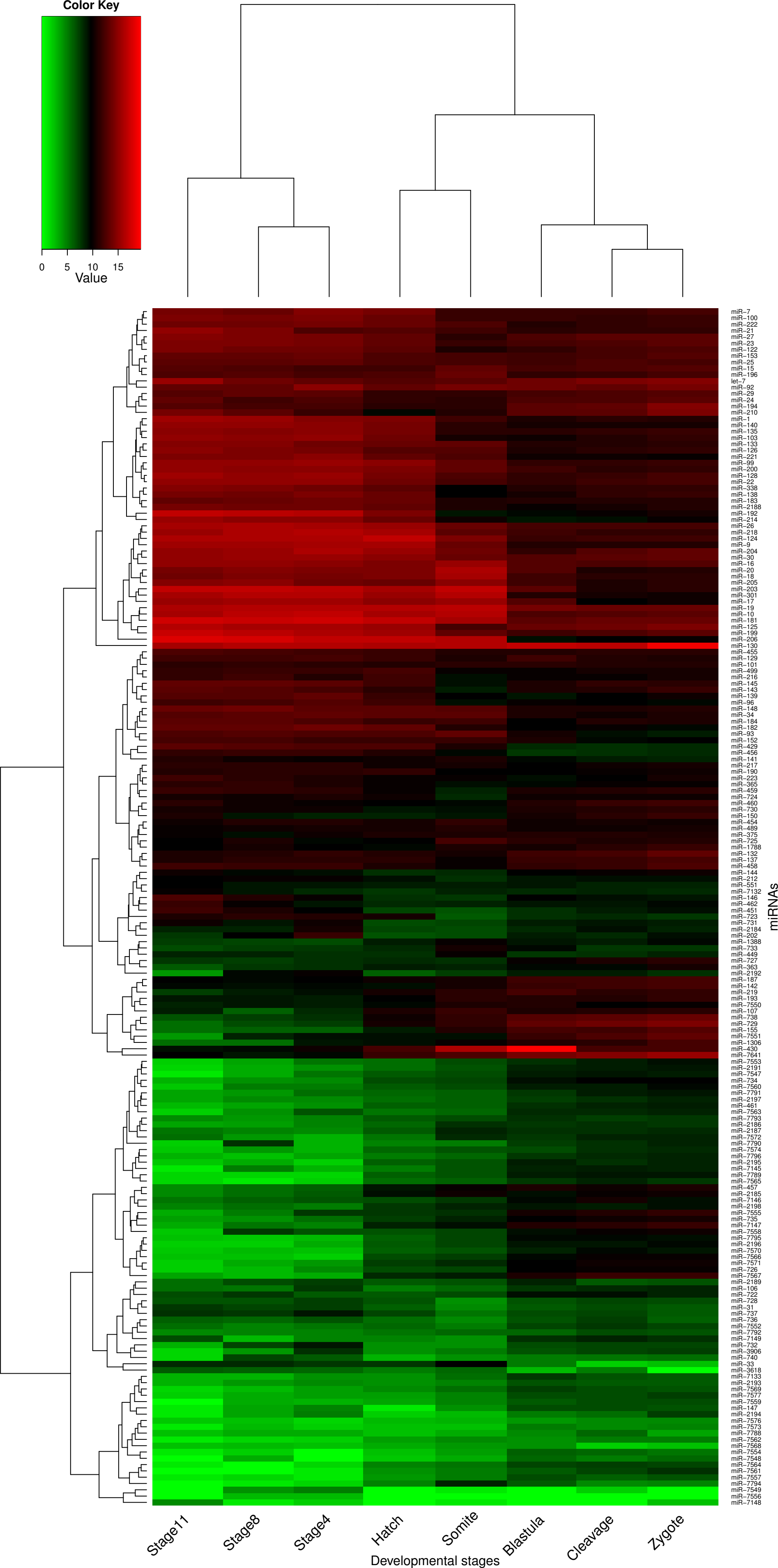

Supplement: Additional file 3: — Heatmap of miRNA expression during Atlantic cod embryonic and larval development (8 developmental stages) at 4°C. High and low expression is marked with red and green, respectively. [file 12864_2015_1503_MOESM3_ESM.pdf]

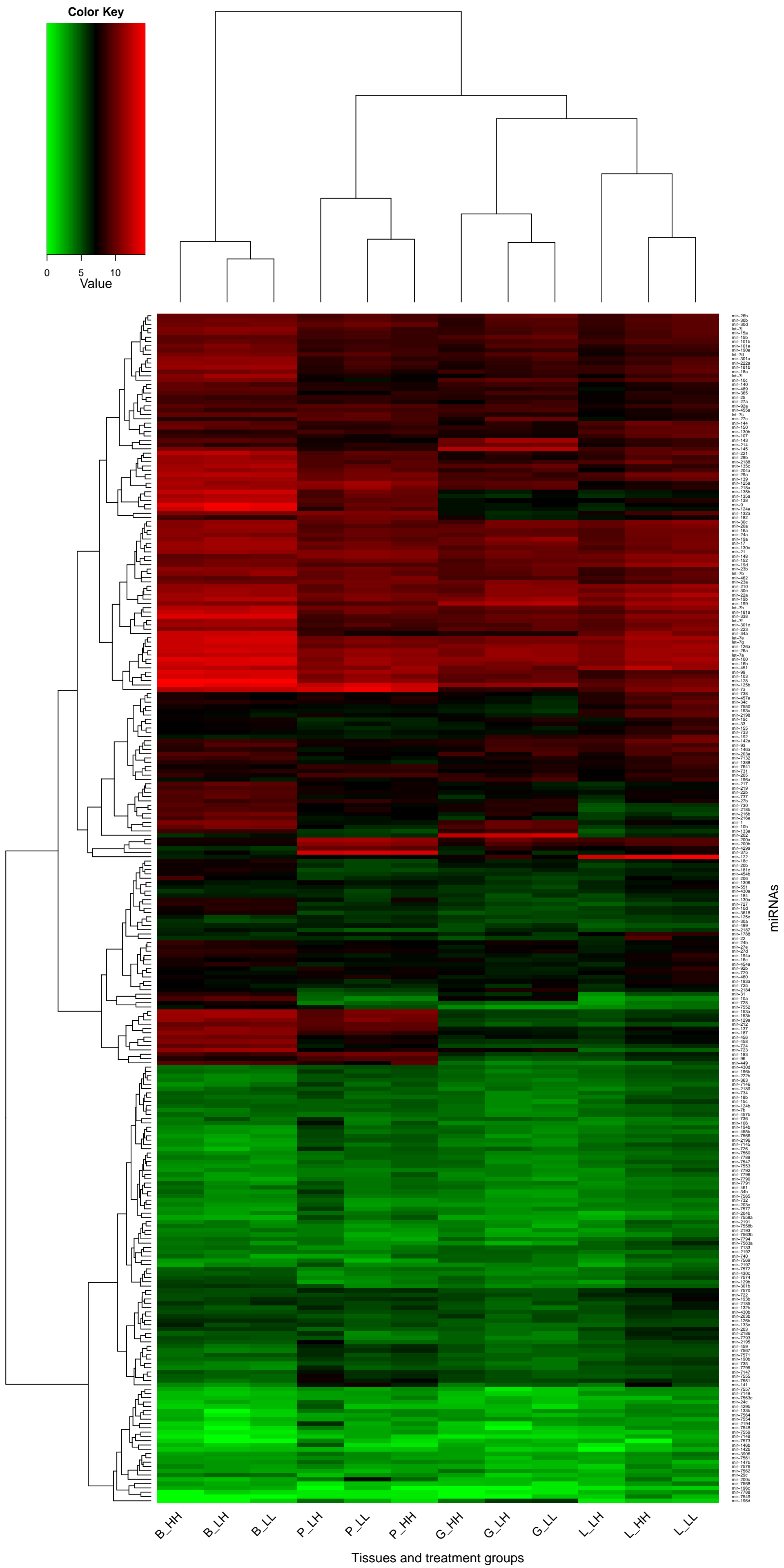

Supplement: Additional file 4: — Heatmap of miRNA expression in 4 organs and tissues of 10 g juvenile Atlantic cod. Fish were reared under 3 temperature regimes (LL, LH and HH). For details of the temperature regimes see Methods. B, P, G, and L stand for brain, pituitary, gonad, and liver, respectively. High and low expression is marked with red and green, respectively. [file 12864_2015_1503_MOESM4_ESM.pdf]

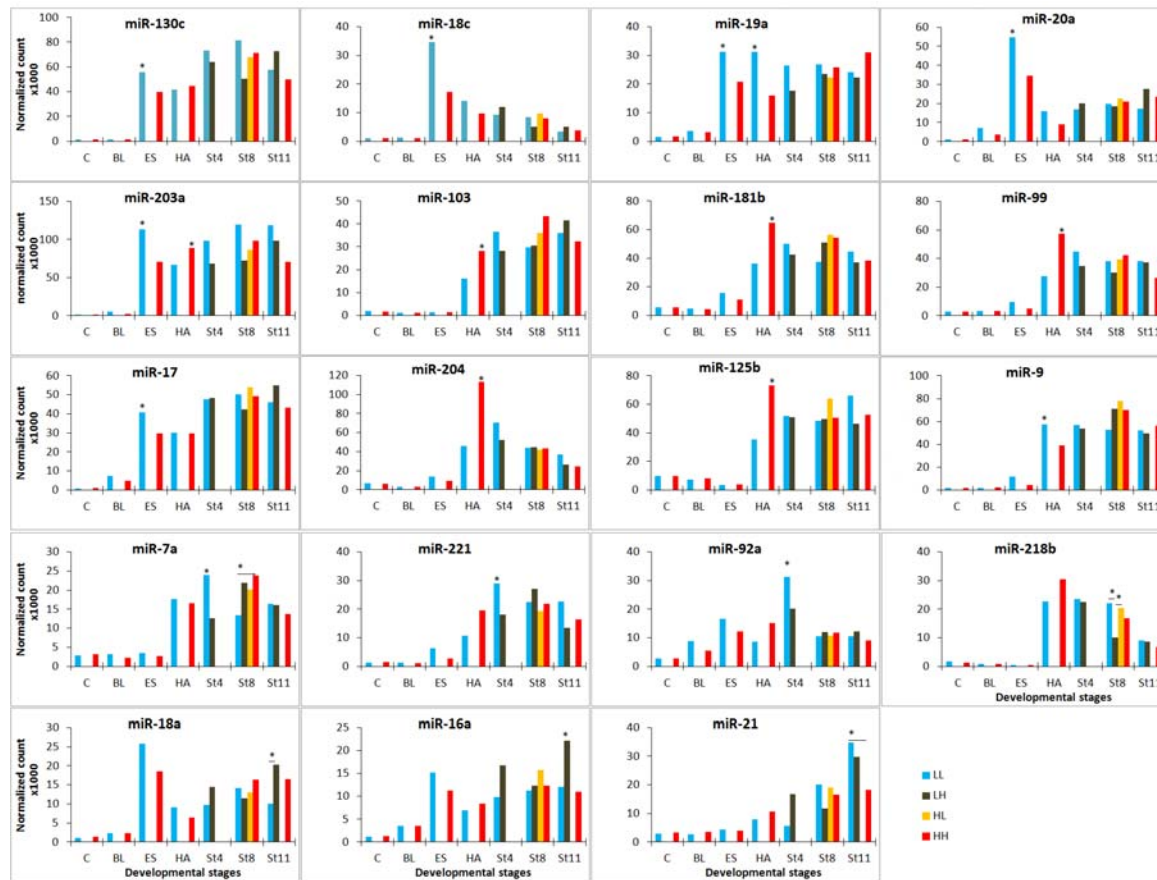

Supplement: Additional file 6: — Differential expression of miRNAs during Atlantic cod embryonic and larval development under four temperature regimes, based on pair-wise comparison between treatments. * indicates significant q-value, which is the probability of differential expression, > 0.99. LL, LH, HL, and HH are temperature regimes described in Methods. C, BL, ES, HA, St4, St8, and St11 stand for cleavage, blastula, early somitogenesis, hatch, stage 4 larvae, stage 8 larvae, and stage 11 larvae developmental stages, respectively. [file 12864_2015_1503_MOESM6_ESM.pdf]

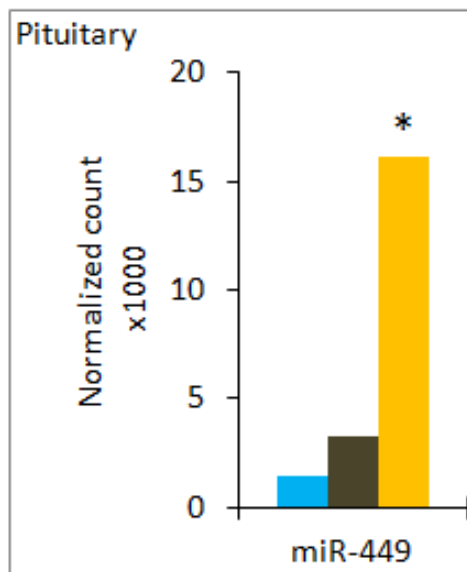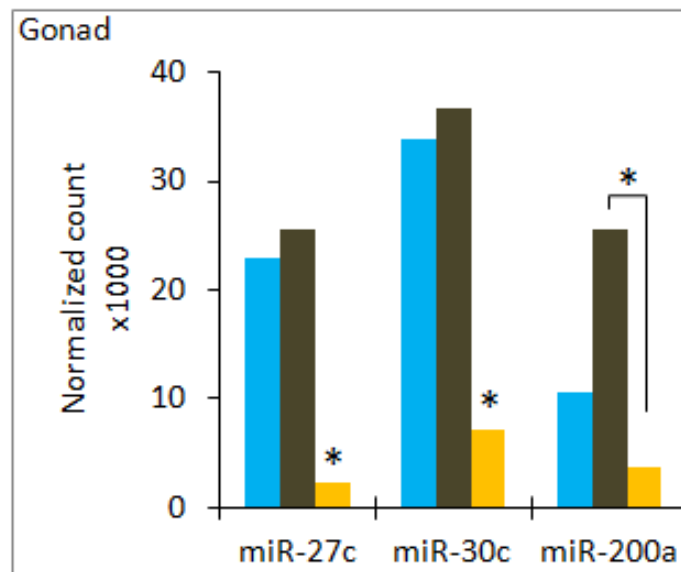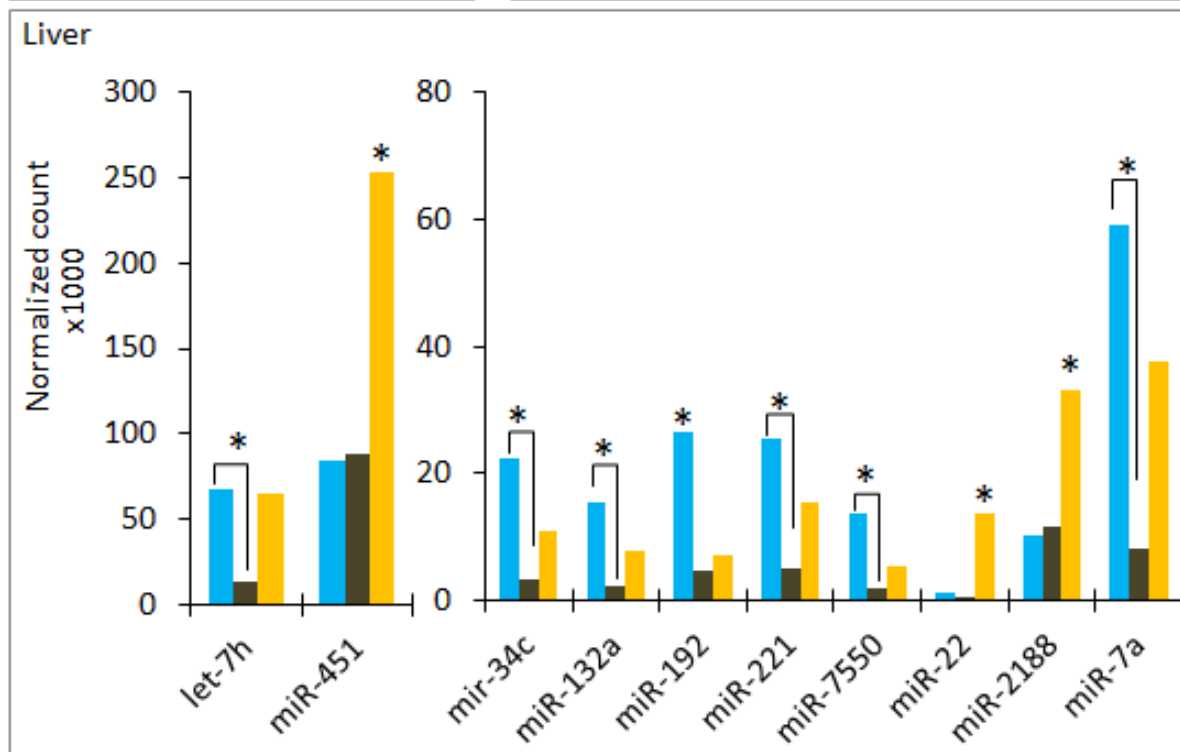

LL LH HH

Supplement: Additional file 7: — Differential expression of miRNAs in pituitary, gonads, and liver of Atlantic cod juveniles reared in three temperature regimes. * stands for significant q-value, which is the probability of differential expression > 0.99. LL, LH, and HH are temperature regimes described in Methods. [file 12864_2015_1503_MOESM7_ESM.pdf]

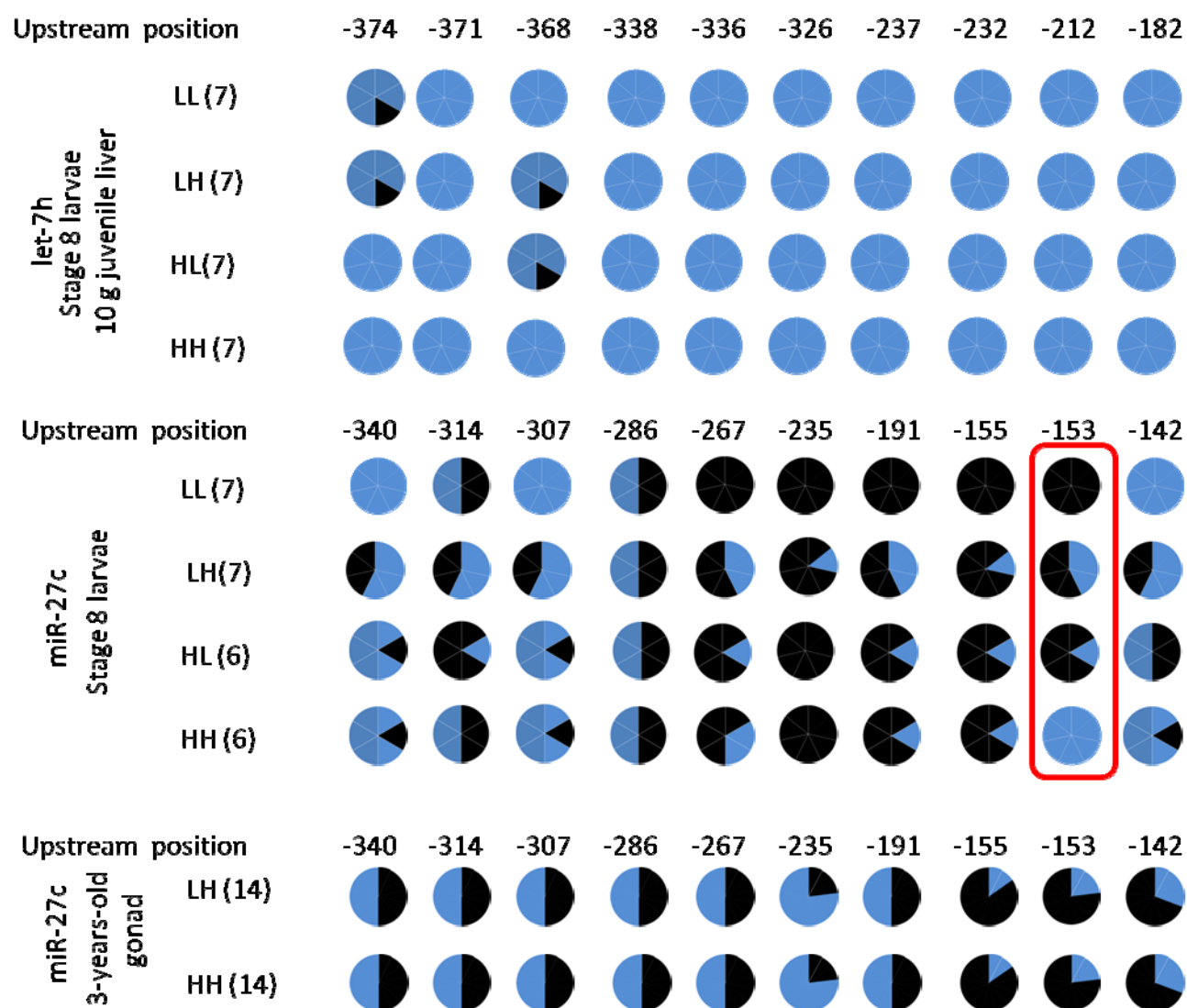

Supplement: Additional file 8: — DNA methylation status at upstream region of miRNA genes in stage 8 larvae, 10 g juvenile liver, and in gonads of adult individuals. The percentage of individuals with methylated (blue) and unmethylated (black) DNA at specific CpG position is displayed. CpG positions are labeled from the start of the pre-miRNA with negative numbers. Numbers of individuals examined in each temperature group are given in brackets. LL, LH, HL, and HH are temperature regimes described in Methods. [file 12864_2015_1503_MOESM8_ESM.pdf]
