# Supplementary material for: Temperature during early development has long-term effects on microRNA expression in Atlantic cod
Source: BMC Genomics. 2015 Apr 17;16(1):305. doi: 10.1186/s12864-015-1503-7 (PMC4403832; doi:10.1186/s12864-015-1503-7)
Supplement: Additional file 9: — Differentially expressed miRNAs during Atlantic cod development and some of the functions of their homologs in other species. [file 12864_2015_1503_MOESM9_ESM.docx]

Table S4 Differentially expressed miRNAs during Atlantic cod development and some of the functions of their homologs in other species.

| Developmental stages | miRNA | Species | Function | References |
| --- | --- | --- | --- | --- |
| Late blastula |  |  |  |  |
|  | miR-130b | Human | Differentiation and proliferation of embryonic neural progenitor cells | ([Gong, Zhang et al. 2013](#_ENREF_18)) |
|  | miR-430 | Zebrafish | Maternal transcript clearance,  PGC guidance,  Nodal signaling balance | ([Giraldez, Mishima et al. 2006](#_ENREF_17))  ([Staton, Knaut et al. 2011](#_ENREF_52))  ([Choi, Giraldez et al. 2007](#_ENREF_8)) |
|  |  |  |  |  |
| Early somites |  |  |  |  |
|  | miR-10b | Zebrafish | Hindbrain patterning | ([Woltering and Durston 2008](#_ENREF_56)) |
|  | miR-17 | Mouse,  Human | Embryonic development  Antiangiogenic activity | ([Foshay and Gallicano 2009](#_ENREF_14))  ([Doebele, Bonauer et al. 2010](#_ENREF_11)) |
|  | miR-19a | Mouse | Cell cycle regulation | ([Qin, Wang et al. 2010](#_ENREF_46)) |
|  | miR-19b | Mouse | Cardiomyocyte differentiaton | ([Qin, Qian et al. 2013](#_ENREF_45)) |
|  | miR-20a | Mouse | Embryonic development  Antiangiogenic activity | ([Foshay and Gallicano 2009](#_ENREF_14))  ([Doebele, Bonauer et al. 2010](#_ENREF_11)) |
|  | miR-181a | Bovine | Maternal transcript (Nmp2) clearance | ([Lingenfelter, Tripurani et al. 2011](#_ENREF_34)) |
|  | miR-203a | Mouse cell | Keratinocyte differentiation and epithelial development | ([Lena, Shalom-Feuerstein et al. 2008](#_ENREF_29)) |
|  | miR-206 | Zebrafish | Cellular movement  Muscle development  Angigenesis | ([Liu, Ning et al. 2012](#_ENREF_35))  ([McCarthy 2008](#_ENREF_38))  ([Stahlhut, Suarez et al. 2012](#_ENREF_51)) |
|  | miR-301c | Nile tilapia | Body growth | ([Huang, Li et al. 2012](#_ENREF_21)) |
|  |  |  |  |  |
| Hatch |  |  |  |  |
|  | miR-9 | Zebrafish | Neurogenesis | ([Coolen, Thieffry et al. 2012](#_ENREF_10)) |
|  | miR-19a | Rat cell | Axonal outgrow | ([Zhang, Ueno et al. 2013](#_ENREF_63)) |
|  | miR-19b | Rat cell | Axonal outgrow | ([Zhang, Ueno et al. 2013](#_ENREF_63)) |
|  | miR-99 | Mammalian cells  Mouse | DNA damage response  Chondrogenesis | ([Mueller, Sun et al. 2013](#_ENREF_39))  ([Yang, Guo et al. 2011](#_ENREF_61)) |
|  | miR-103 | Porcine  Mouse | Adipocyte differentiation Intestinal cryptic cell proliferation | ([Li, Li et al. 2011](#_ENREF_30))  ([Liao and Lönnerdal 2010](#_ENREF_33)) |
|  | miR-124 | Zebrafish | Neurogenesis | ([Kapsimali, Kloosterman et al. 2007](#_ENREF_22)) |
|  | miR-125b | Human  Zebrafish | Neural differentiation | ([Le, Teh et al. 2009](#_ENREF_27))  ([Le, Teh et al. 2010](#_ENREF_26)) |
|  | miR-181a | Mouse,  Zebrafish | Thymocyte development  Lymphogenesis | ([Neilson, Zheng et al. 2007](#_ENREF_40))  ([Dunworth, Cardona-Costa et al. 2013](#_ENREF_12)) |
|  | miR-181b | Mouse | Thymocyte development | ([Neilson, Zheng et al. 2007](#_ENREF_40)) |
|  | miR-192 | Nile tilapia | Body growth | ([Huang, Li et al. 2012](#_ENREF_21)) |
|  | miR-203a | Mouse | Keratinocyte differentiation and epithelial development  Digestive tract organogenesis | ([Lena, Shalom-Feuerstein et al. 2008](#_ENREF_29))  ([Kim, Woo et al. 2011](#_ENREF_24)) |
|  | miR-204 | Mouse and zebrafish | Lens development | ([Conte, Carrella et al. 2010](#_ENREF_9); [Ramachandran, Fausett et al. 2010](#_ENREF_47); [Shaham, Gueta et al. 2013](#_ENREF_49)) |
|  | miR-205 | Cell lines | Epithelial to mescenchymal transition | ([Gregory, Bert et al. 2008](#_ENREF_19)) |
|  |  |  |  |  |
|  | miR-214 | Common carp | Skeletal muscle development | ([Yan, Ding et al. 2012](#_ENREF_60)) |
|  | miR-218a | Zebrafish  Nile tilapia | Neuronal development  Skeletal muscle growth | ([Kapsimali, Kloosterman et al. 2007](#_ENREF_22))  ([Huang, Li et al. 2012](#_ENREF_21)) |
|  | miR-301c | Nile tilapia | Body growth | ([Huang, Li et al. 2012](#_ENREF_21)) |
|  |  |  |  |  |
| Stage 4 |  |  |  |  |
|  | miR-7a | Mouse  Zebrafish | Digestive tract organogenesis  Endocrine development | ([Kim, Woo et al. 2011](#_ENREF_24))  ([Wienholds, Kloosterman et al. 2005](#_ENREF_55)) |
|  | miR-10b | Zebrafish  Human | Neural development  Adipogenesis | ([Wienholds, Kloosterman et al. 2005](#_ENREF_55))  ([Guo, Mo et al. 2012](#_ENREF_20)) |
|  | miR-92a | Rat  Mouse  Zebrafish | Axonal outgrow  Angiogenesis  Chondrogenesis | ([Zhang, Ueno et al. 2013](#_ENREF_63))  ([Bonauer, Carmona et al. 2009](#_ENREF_4))  ([Ning, Liu et al. 2013](#_ENREF_42)) |
|  | miR-124 | Zebrafish  Medaka | Neuronal development | ([Kapsimali, Kloosterman et al. 2007](#_ENREF_22))  ([Kato, Kusakabe et al. 2013](#_ENREF_23)) |
|  | miR-130b | Mouse | Proliferation of neural cells | ([Gong, Zhang et al. 2013](#_ENREF_18)) |
|  | miR-221 | Zebrafish | Angiogenesis | ([Nicoli, Knyphausen et al. 2012](#_ENREF_41)) |
|  |  |  |  |  |
| Stage 8 |  |  |  |  |
|  | miR-7a | Mouse  Zebrafish | Digestive tract organogenesis  Endocrine development | ([Kim, Woo et al. 2011](#_ENREF_24))  ([Wienholds, Kloosterman et al. 2005](#_ENREF_55)) |
|  | miR-10b | Zebrafish  Human | Neural development  Adipogenesis | ([Wienholds, Kloosterman et al. 2005](#_ENREF_55))  ([Guo, Mo et al. 2012](#_ENREF_20)) |
|  | miR-124 | Mouse | Osetoclastogenesis | ([Lee, Kim et al. 2013](#_ENREF_28)) |
|  | miR-130b |  |  |  |
|  | miR-192 | Nile tilapia  Asian seabass | Body growth  Immunological response | ([Huang, Li et al. 2012](#_ENREF_21))  ([Xia, He et al. 2011](#_ENREF_58)) |
|  | miR-205 |  |  |  |
|  | miR-206 | Mouse  Nile tilapia | Muscle differentiation  Muscle growth | ([Anderson, Catoe et al. 2006](#_ENREF_2))  ([Yan, Zhu et al. 2013](#_ENREF_59)) |
|  | miR-218a | Zebrafish  Nile tilapia | Neuronal development  Skeletal muscle growth | ([Kapsimali, Kloosterman et al. 2007](#_ENREF_22))  ([Huang, Li et al. 2012](#_ENREF_21)) |
|  |  |  |  |  |
| Stage 11 |  |  |  |  |
|  | miR-16a | Zebrafish | Thymocyte development  Olfactory system | ([Neilson, Zheng et al. 2007](#_ENREF_40))  ([Wang, Bammler et al. 2013](#_ENREF_54)) |
|  | miR-18a | Rat cell  Zebrafish  Mouse | Axonal outgrow  Inner ear development  Pancreatic development | ([Zhang, Ueno et al. 2013](#_ENREF_63))  ([Friedman, Dror et al. 2009](#_ENREF_15))  ([An, Yang et al. 2009](#_ENREF_1)) |
|  | miR-21 | Common carp  Asian seabass | Thymocyte development  Skeletal muscle development  Immune response | ([Neilson, Zheng et al. 2007](#_ENREF_40))  ([Yan, Ding et al. 2012](#_ENREF_60))  ([Xia, He et al. 2011](#_ENREF_58)) |
|  | miR-130b | Human | Cell growth and renewal | ([Ma, Tang et al. 2010](#_ENREF_36)) |
|  |  |  |  |  |
|  |  |  |  |  |

| Tissue  /organ | miRNA | Species | Function | References |
| --- | --- | --- | --- | --- |
| Pituitary |  |  |  |  |
|  | miR-449 | Xenopus and human | Multiciliogenesis  Cell cycle | ([Marcet, Chevalier et al. 2011](#_ENREF_37))  ([Yang, Feng et al. 2009](#_ENREF_62)) |
| Gonad |  |  |  |  |
|  | miR-27c | Zebrafish | Vessel formation  Endothelial cell repulsion | ([Urbich, Kaluza et al. 2012](#_ENREF_53)) miR-27a/b |
|  | miR-30c | Zebrafish | Angiogenesis | ([Bridge, Monteiro et al. 2012](#_ENREF_6)) |
|  | miR-200a | Human | epithelial-mesenchymal transition | ([Bracken, Gregory et al. 2008](#_ENREF_5)) |
| Liver |  |  |  |  |
|  | let-7h | Mouse | Glucose homeostasis and fat metabolism | ([Frost and Olson 2011](#_ENREF_16)) |
|  | miR-7a | Human | Cell cycle | ([Sanchez, Gallagher et al. 2013](#_ENREF_48)) |
|  | miR-22 | Rat | Metabolic pathways  Cell cycle | ([Koturbash, Melnyk et al. 2013](#_ENREF_25))  ([Pandey and Picard 2009](#_ENREF_43); [Bar and Dikstein 2010](#_ENREF_3)) |
|  | miR-34c | Rat | DNA damage control  Fatty acid metabolism | ([Cannell, Kong et al. 2010](#_ENREF_7))  ([Li, Chen et al. 2011](#_ENREF_32)) |
|  | miR-132a | Human | Immune response | ([Estep, Armistead et al. 2010](#_ENREF_13)) |
|  | miR-192 | Mammalian Cell  Asian seabass and zebrafish | Thymocyte development  Immune response | ([Neilson, Zheng et al. 2007](#_ENREF_40))  ([Xia, He et al. 2011](#_ENREF_58); [Wu, Pan et al. 2012](#_ENREF_57)) |
|  | miR-221 | Mammalian cell | Thymocyte development | ([Neilson, Zheng et al. 2007](#_ENREF_40)) |
|  | miR-451 | Human  zebrafish | Cell cycle  Erythroid maturation | ([Pase, Layton et al. 2009](#_ENREF_44); [Li, Zeng et al. 2013](#_ENREF_31)) |
|  | miR-2188 | Zebrafish | Vessel development | ([Soares, Reverendo et al. 2012](#_ENREF_50)) |

An, Y., Y. K. Yang, et al. (2009). "Identification of micro RNAs regulating ptfla expression in mouse pancreas development." Progress in Biochemistry and Biophysics **36**(12): 1607-1612.

Anderson, C., H. Catoe, et al. (2006). "MIR-206 regulates connexin43 expression during skeletal muscle development." Nucleic Acids Research **34**(20): 5863-5871.

Bar, N. and R. Dikstein (2010). "miR-22 Forms a Regulatory Loop in PTEN/AKT Pathway and Modulates Signaling Kinetics." PLoS ONE **5**(5): e10859.

Bonauer, A., G. Carmona, et al. (2009). "MicroRNA-92a Controls Angiogenesis and Functional Recovery of Ischemic Tissues in Mice." Science **324**(5935): 1710-1713.

Bracken, C. P., P. A. Gregory, et al. (2008). "A double-negative feedback loop between ZEB1-SIP1 and the microRNA-200 family regulates epithelial-mesenchymal transition." Cancer Research **68**(19): 7846-7854.

Bridge, G., R. Monteiro, et al. (2012). "The microRNA-30 family targets DLL4 to modulate endothelial cell behavior during angiogenesis." Blood **120**(25): 5063-5072.

Cannell, I. G., Y. W. Kong, et al. (2010). "p38 MAPK/MK2-mediated induction of miR-34c following DNA damage prevents Myc-dependent DNA replication." Proceedings of the National Academy of Sciences of the United States of America **107**(12): 5375-5380.

Choi, W.-Y., A. J. Giraldez, et al. (2007). "Target Protectors Reveal Dampening and Balancing of Nodal Agonist and Antagonist by miR-430." Science **318**(5848): 271-274.

Conte, I., S. Carrella, et al. (2010). "miR-204 is required for lens and retinal development via Meis2 targeting." Proceedings of the National Academy of Sciences **107**(35): 15491-15496.

Coolen, M., D. Thieffry, et al. (2012). "miR-9 Controls the Timing of Neurogenesis through the Direct Inhibition of Antagonistic Factors." Developmental Cell **22**(5): 1052-1064.

Doebele, C., A. Bonauer, et al. (2010). "Members of the microRNA-17-92 cluster exhibit a cell-intrinsic antiangiogenic function in endothelial cells." Blood **115**(23): 4944-4950.

Dunworth, W. P., J. Cardona-Costa, et al. (2013). "Bone Morphogenetic Protein 2 Signaling Negatively Modulates Lymphatic Development in Vertebrate Embryos." Circulation Research.

Estep, M., D. Armistead, et al. (2010). "Differential expression of miRNAs in the visceral adipose tissue of patients with non-alcoholic fatty liver disease." Alimentary Pharmacology & Therapeutics **32**(3): 487-497.

Foshay, K. M. and G. I. Gallicano (2009). "miR-17 family miRNAs are expressed during early mammalian development and regulate stem cell differentiation." Developmental Biology **326**(2): 431-443.

Friedman, L. M., A. A. Dror, et al. (2009). "MicroRNAs are essential for development and function of inner ear hair cells in vertebrates." Proceedings of the National Academy of Sciences **106**(19): 7915-7920.

Frost, R. J. A. and E. N. Olson (2011). "Control of glucose homeostasis and insulin sensitivity by the Let-7 family of microRNAs." Proceedings of the National Academy of Sciences of the United States of America **108**(52): 21075-21080.

Giraldez, A. J., Y. Mishima, et al. (2006). "Zebrafish MiR-430 promotes deadenylation and clearance of maternal mRNAs." Science **312**(5770): 75-79.

Gong, X., K. Zhang, et al. (2013). "MicroRNA-130b targets Fmr1 and regulates embryonic neural progenitor cell proliferation and differentiation." Biochemical and Biophysical Research Communications **439**(4): 493-500.

Gregory, P. A., A. G. Bert, et al. (2008). "The miR-200 family and miR-205 regulate epithelial to mesenchymal transition by targeting ZEB1 and SIP1." Nature Cell Biology **10**(5): 593-601.

Guo, Y., D. Mo, et al. (2012). "MicroRNAome Comparison between Intramuscular and Subcutaneous Vascular Stem Cell Adipogenesis." PLoS ONE **7**(9): e45410.

Huang, C. W., Y. H. Li, et al. (2012). "Differential expression patterns of growth-related microRNAs in the skeletal muscle of Nile tilapia (*Oreochromis niloticus*)." Journal of Animal Science **90**(12): 4266-4279.

Kapsimali, M., W. P. Kloosterman, et al. (2007). "MicroRNAs show a wide diversity of expression profiles in the developing and mature central nervous system." Genome Biology **8**(8): R173.

Kato, Y., R. Kusakabe, et al. (2013). "MiR-124 is Involved in Post-transcriptional Regulation of Polypyrimidine Tract Binding Protein 1 (PTBP1) During Neural Development in the Medaka, Oryzias latipes." Zoological Science **30**(11): 891-900.

Kim, B. M., J. Woo, et al. (2011). "Regulation of mouse stomach development and Barx1 expression by specific microRNAs." Development **138**(6): 1081-1086.

Koturbash, I., S. Melnyk, et al. (2013). "Role of epigenetic and miR-22 and miR-29b alterations in the downregulation of Mat1a and Mthfr genes in early preneoplastic livers in rats induced by 2-acetylaminofluorene." Molecular Carcinogenesis **52**(4): 318-327.

Le, M. T. N., C. Teh, et al. (2010). "Function of miR-125b in zebrafish neurogenesis." World Academy of Science, Engineering and Technology **62**: 635-640.

Le, M. T. N., C. Teh, et al. (2009). "MicroRNA-125b is a novel negative regulator of p53." Genes & Development **23**(7): 862-876.

Lee, Y., H. J. Kim, et al. (2013). "MicroRNA-124 regulates osteoclast differentiation." Bone **56**(2): 383-389.

Lena, A. M., R. Shalom-Feuerstein, et al. (2008). "miR-203 represses /`stemness/' by repressing [Delta]Np63." Cell Death Differ **15**(7): 1187-1195.

Li, G., Y. Li, et al. (2011). "MicroRNA identity and abundance in developing swine adipose tissue as determined by solexa sequencing." Journal of Cellular Biochemistry **112**(5): 1318-1328.

Li, H. P., X. C. Zeng, et al. (2013). "miR-451 inhibits cell proliferation in human hepatocellular carcinoma through direct suppression of IKK-beta." Carcinogenesis **34**(11): 2443-2451.

Li, W.-Q., C. Chen, et al. (2011). "The rno-miR-34 family is upregulated and targets ACSL1 in dimethylnitrosamine-induced hepatic fibrosis in rats." FEBS Journal **278**(9): 1522-1532.

Liao, Y. and B. Lönnerdal (2010). "Global MicroRNA characterization reveals that miR-103 is involved in IGF-1 stimulated mouse intestinal cell proliferation." PLoS ONE **5**(9).

Lingenfelter, B. M., S. K. Tripurani, et al. (2011). "Molecular cloning and expression of bovine nucleoplasmin 2 (NPM2): A maternal effect gene regulated by miR-181a." Reproductive Biology and Endocrinology **9**.

Liu, X., G. Ning, et al. (2012). "MicroRNA-206 regulates cell movements during zebrafish gastrulation by targeting prickle1a and regulating c-Jun N-terminal kinase 2 phosphorylation." Molecular and Cellular Biology **32**(14): 2934-2942.

Ma, S., K. H. Tang, et al. (2010). "miR-130b Promotes CD133+ Liver Tumor-Initiating Cell Growth and Self-Renewal via Tumor Protein 53-Induced Nuclear Protein 1." Cell Stem Cell **7**(6): 694-707.

Marcet, B., B. Chevalier, et al. (2011). "Control of vertebrate multiciliogenesis by miR-449 through direct repression of the Delta/Notch pathway." Nature Cell Biology **13**(6): 693-701.

McCarthy, J. J. (2008). "MicroRNA-206: The skeletal muscle-specific myomiR." Biochimica Et Biophysica Acta-Gene Regulatory Mechanisms **1779**(11): 682-691.

Mueller, A. C., D. Sun, et al. (2013). "The miR-99 family regulates the DNA damage response through its target SNF2H." Oncogene **32**(9): 1164-1172.

Neilson, J. R., G. X. Y. Zheng, et al. (2007). "Dynamic regulation of miRNA expression in ordered stages of cellular development." Genes & Development **21**(5): 578-589.

Nicoli, S., C.-P. Knyphausen, et al. (2012). "miR-221 Is Required for Endothelial Tip Cell Behaviors during Vascular Development." Developmental Cell **22**(2): 418-429.

Ning, G., X. Liu, et al. (2013). "MicroRNA-92a Upholds Bmp Signaling by Targeting noggin3 during Pharyngeal Cartilage Formation." Developmental Cell **24**(3): 283-295.

Pandey, D. P. and D. Picard (2009). "miR-22 Inhibits Estrogen Signaling by Directly Targeting the Estrogen Receptor α mRNA." Molecular and Cellular Biology **29**(13): 3783-3790.

Pase, L., J. E. Layton, et al. (2009). "miR-451 regulates zebrafish erythroid maturation in vivo via its target gata2." Blood **113**(8): 1794-1804.

Qin, D. N., L. Qian, et al. (2013). "Effects of miR-19b Overexpression on Proliferation, Differentiation, Apoptosis and Wnt/β-Catenin Signaling Pathway in P19 Cell Model of Cardiac Differentiation In Vitro." Cell Biochemistry and Biophysics **66**(3): 709-722.

Qin, X., X. Wang, et al. (2010). "MicroRNA-19a mediates the suppressive effect of laminar flow on cyclin D1 expression in human umbilical vein endothelial cells." Proceedings of the National Academy of Sciences **107**(7): 3240-3244.

Ramachandran, R., B. V. Fausett, et al. (2010). "Ascl1a regulates Muller glia dedifferentiation and retinal regeneration through a Lin-28-dependent, let-7 microRNA signalling pathway." Nature Cell Biology **12**(11): 1101-U1106.

Sanchez, N., M. Gallagher, et al. (2013). "MiR-7 Triggers Cell Cycle Arrest at the G1/S Transition by Targeting Multiple Genes Including Skp2 and Psme3." PLoS ONE **8**(6): e65671.

Shaham, O., K. Gueta, et al. (2013). "Pax6 Regulates Gene Expression in the Vertebrate Lens through miR-204." PLoS Genetics **9**(3): e1003357.

Soares, A. R., M. Reverendo, et al. (2012). "Dre-miR-2188 Targets Nrp2a and Mediates Proper Intersegmental Vessel Development in Zebrafish Embryos." PLoS ONE **7**(6).

Stahlhut, C., Y. Suarez, et al. (2012). "miR-1 and miR-206 regulate angiogenesis by modulating VegfA expression in zebrafish." Development **139**(23): 4356-4364.

Staton, A. A., H. Knaut, et al. (2011). "miRNA regulation of Sdf1 chemokine signaling provides genetic robustness to germ cell migration." Nature Genetics **43**(3): 204-U245.

Urbich, C., D. Kaluza, et al. (2012). "MicroRNA-27a/b controls endothelial cell repulsion and angiogenesis by targeting semaphorin 6A." Blood **119**(6): 1607-1616.

Wang, L., T. K. Bammler, et al. (2013). "Copper-Induced Deregulation of microRNA Expression in the Zebrafish Olfactory System." Environmental Science & Technology **47**(13): 7466-7474.

Wienholds, E., W. P. Kloosterman, et al. (2005). "MicroRNA expression in zebrafish embryonic development." Science **309**(5732): 310-311.

Woltering, J. M. and A. J. Durston (2008). "MiR-10 Represses HoxB1a and HoxB3a in Zebrafish." PLoS ONE **3**(1): 13.

Wu, T. H., C. Y. Pan, et al. (2012). "In vivo screening of zebrafish microRNA responses to bacterial infection and their possible roles in regulating immune response genes after lipopolysaccharide stimulation." Fish Physiology and Biochemistry **38**(5): 1299-1310.

Xia, J. H., X. P. He, et al. (2011). "Identification and Characterization of 63 MicroRNAs in the Asian Seabass *Lates calcarifer*." PLoS ONE **6**(3): 11.

Yan, B., C.-D. Zhu, et al. (2013). "miR-206 regulates the growth of the teleost tilapia (*Oreochromis niloticus*) through the modulation of IGF-1 gene expression." Journal of Experimental Biology **216**(7): 1265-1269.

Yan, X. C., L. Ding, et al. (2012). "Identification and Profiling of MicroRNAs from Skeletal Muscle of the Common Carp." PLoS ONE **7**(1): e30925.

Yang, B., H. F. Guo, et al. (2011). "The microRNA expression profiles of mouse mesenchymal stem cell during chondrogenic differentiation." Bmb Reports **44**(1): 28-33.

Yang, X., M. Feng, et al. (2009). "miR-449a and miR-449b are direct transcriptional targets of E2F1 and negatively regulate pRb-E2F1 activity through a feedback loop by targeting CDK6 and CDC25A." Genes & Development **23**(20): 2388-2393.

Zhang, Y., Y. Ueno, et al. (2013). "The MicroRNA-17-92 cluster enhances axonal outgrowth in embryonic cortical neurons." Journal of Neuroscience **33**(16): 6885-6894.
