# Supplementary material for: Temperature during early development has long-term effects on microRNA expression in Atlantic cod
Source: BMC Genomics. 2015 Apr 17;16(1):305. doi: 10.1186/s12864-015-1503-7 (PMC4403832; doi:10.1186/s12864-015-1503-7)
Supplement: Additional file 10: — Primers. Top table: primers used to amplify the bisulfite treated and untreated DNA. Bottom table: LNA primers used for rt-qPCR and their efficiency. [file 12864_2015_1503_MOESM10_ESM.docx]

Table S1a. Sequences of primers used to amplify the bisulfite treated and untreated DNA.

| Primer name | Bisufite (5' - 3') | Non-bisulfite (5' - 3') |
| --- | --- | --- |
| let-7h-1F | AATGGYGTAGAGAATATTGTAGGTAG | AATGGCGCAGAGAACACTGCAGGTAG |
| let-7h-1R | AAACCTCCRCTATTCAATATTTACAAAC | CGTGCTGTGGGATGAGGTAGTAGTTTGT |
| miR-124F | TAAATATTYGGTGTTTTTTTTTGGGGGG | CAAACACTCGGTGTTTTTTTTTGGGGGG |
| miR-124R | AAACATCRCAATAAACCACATCTTCCC | GAGCATCGCAGTGGACCACATCTTCCC |
| miR-132aF | TTAGTTYGATATTATTTTAGGGGGGTG | TCAGTCCGACACTACCTCAGGGGGGTG |
| miR-132aR | TTAAAAACACTTTTTTCTTCCCTCTCTC | TTAGAGACACTTTTTTCTTCCCTCTCTC |
| miR-16aF | GGAAGTAGTTAATATATGATGGTGGGG | GGAAGCAGCTAATACATGATGGTGGGG |
| miR-16aR | ACAAACATTCTCTCTACACTTAAACAC | GCAAACATTCTCTCTGCACTTAAACAC |
| miR-200aF | GTATTGAGGATTGTGTTTATATTGGGTG | CCAATATAAACACAATCCTCAATACAAC |
| miR-200aR | CTAAAAACCRATTCTTCCAAATAACACC | CTGGGAGCCGGTTCTTCCAGATGACACC |
| miR-205F | AATGATYGGTTGAGGGTAGTTTGATTG | AATGATCGGTTGAGGGCAGTCTGATTG |
| miR-205R | CCAAAACCRACCAACAAACCAAAAAATC | CCAAAACCGACCAACAAGCCAGGAAGTC |
| miR-2188F | ATATTAATYGGTTTAGGGTTGATGAGG | ACATTAATCGGTCTAGGGCTGATGAGG |
| miR-2188R | CCTACCACCCTCCTCCCCTCCCTC | CCTGCCACCCTCCTCCCCTCCCTC |
| miR-221F | TTGTTTGTYGGTTTTTTTTTGTTTTTGG | TTGTCTGCCGGCCCCTCTCTGTCCTTGG |
| miR-221R | AAACACRATATACATCCCATCTTCC | GGACACGGTGTGCATCCCATCTTCC |
| miR-27cF | YGTAATTGAGGTTTATTTAGTTGGTGG | CGTAATTGAGGCCCACTCAGTTGGTGG |
| miR-27cR | ATTTCCCAACATCCCAAATTATTACTTC | ATTTCCCAACATCCCAAATTATTACTTC |
| miR-30cF | TTAGAGGAYGTTTGGTTTTAAATTTTTG | TCAGAGGACGCTTGGTCTTAAATTCTTG |
| miR-30cR | CACACCRTCTAAAACACAAATATTTAAC | CACACCGTCTAAGGCACAAGTGTTTAGC |
| miR-92aF | TTTGYGTAGTTGTTTTGTAAGAAAATTG | TTTGCGTAGTTGCTCTGCAAGAAAATTG |
| miR-92aR | AACTAAAAACAAACCAACAAAACTAACC | AGCTGAAAGCAAACCAGCAAAACTAACC |

Table S1b. LNA primers used to amplify mature miRNAs and their efficiency.

| miRNA name | Sequence (5’ to 3’) | Primers efficiency (%) |
| --- | --- | --- |
| miR-7 | UGGAAGACUAGUGAUUUUGUUGUU | No |
| miR-122-5p | UGGAGUGUGACAAUGGUGUUUG | 96.7 |
| miR-192 | AUGACCUAUGAAUUGACAGCC | 96.76 |
| miR-221 | AGCUACAUUGUCUGCUGGGUUU | 90.84 |
| miR-451 | AAACCGUUACCAUUACUGAGUU | 97.19 |
